# Regulatory structural variations upstream of a NAC transcription factor control fruit maturity timing in peach

**DOI:** 10.1093/jxb/erag210

**Published:** 2026-04-29

**Authors:** Alessandro Giulio Tagliabue, James Friel, Remo Chiozzotto, Irina Baccichet, Cassia da Silva Linge, Elisa Calastri, Gianluca Biffi, Nathalia Zaracho, Stefano Gattolin, Sabrina Micali, Iban Eduardo, Daniele Bassi, Laura Rossini, Marco Cirilli

**Affiliations:** Department of Agricultural and Environmental Sciences (DISAA), University of Milan, Milan 20133, Italy; Department of Agricultural and Environmental Sciences (DISAA), University of Milan, Milan 20133, Italy; Department of Agricultural and Environmental Sciences (DISAA), University of Milan, Milan 20133, Italy; Department of Agricultural and Environmental Sciences (DISAA), University of Milan, Milan 20133, Italy; Department of Agricultural and Environmental Sciences (DISAA), University of Milan, Milan 20133, Italy; Department of Agricultural and Environmental Sciences (DISAA), University of Milan, Milan 20133, Italy; Department of Agricultural and Environmental Sciences (DISAA), University of Milan, Milan 20133, Italy; Centre for Research in Agricultural Genomics (CRAG) CSIC-IRTA-UAB-UB, Campus UAB, Edifici CRAG, Cerdanyola del Vallès (Bellaterra), Barcelona 08193, Spain; Institute of Agricultural Biology and Biotechnology (IBBA), CNR-National Research Council of Italy, Milan 20133, Italy; Consiglio per la Ricerca in Agricoltura e L’analisi Dell’Economia Agraria, Research Centre for Olive, Fruit, and Citrus Crops, Rome 00134, Italy; Centre for Research in Agricultural Genomics (CRAG) CSIC-IRTA-UAB-UB, Campus UAB, Edifici CRAG, Cerdanyola del Vallès (Bellaterra), Barcelona 08193, Spain; Department of Agricultural and Environmental Sciences (DISAA), University of Milan, Milan 20133, Italy; Department of Agricultural and Environmental Sciences (DISAA), University of Milan, Milan 20133, Italy; Department of Agricultural and Environmental Sciences (DISAA), University of Milan, Milan 20133, Italy; Università degli Studi di Padova, Italy

**Keywords:** Fruit trees, maturity date, peach, QTL, ripening, structural variants

## Abstract

Fruit maturity date is a key developmental trait in fruit trees, as it determines the timing of when fruits enter the ripening phase, thereby shaping the harvest calendar and fruit quality. In peach, ripening time varies over several months, yet the genetic and regulatory basis underlying this extensive phenological diversity has remained largely unresolved. Here, we dissect the long-standing chromosome 4 maturity date locus (*qMD4.1*) and show that a multi-allelic series of structural variants in the upstream regulatory region of the transcription factor NAC1 explains most of the observed variation in ripening time in cultivated peach. Across multiple independent populations and diverse genetic backgrounds, these variants (collectively termed *Md*) stratify maturity date from ultra-early to late cultivars with largely additive and dosage-dependent effects. Using recombinant-based genetic dissection, long-read sequencing, transcriptomic analyses, and epigenomic data integration, we show that *Md* alleles are consistently associated with allele-dependent shifts in the temporal dynamics of *NAC1* transcript accumulation, and that the affected region overlaps with chromatin features characteristic of regulatory activity. By resolving the genetic architecture of a major phenological locus, our results support a central role for *cis*-regulatory structural variation in modulating developmental timing in peach and provide biologically interpretable markers for predicting maturity date. More broadly, this work illustrates how non-coding structural variation can contribute to variation in fruit developmental trajectories, with implications for breeding strategies aimed at modulating harvest windows in peach and other stone fruit species.

## Teaser

Structural changes in DNA upstream of a key gene (*NAC1*) control when peaches ripen. These variants shift gene activation timing, explaining early or late harvests and enabling breeders to predict fruit maturity.

## Introduction

Across the genus *Prunus*, differences in fruit maturation timing underlie much of the agronomic diversity observed among stone fruit cultivars, influencing adaptation, harvest scheduling, and breeding decisions. Understanding the genetic and regulatory basis of these differences is therefore central to fruit biology and improvement.

Peach [*P. persica* (L.) Batsch] is the reference species for stone fruits due to its economic value, small genome, low heterozygosity, and close phylogenetic relationship with other major *Prunus* crops (Shulaev *et al*., 2008). As a climacteric drupe, peach undergoes a well-defined developmental and ripening program, making it an excellent model for investigating the genetic regulation of fruit maturation. Fruit development in peach typically follows the characteristic double-sigmoid pattern of climacteric drupes, in which coordinated physiological, hormonal, and molecular events determine final size, flesh texture, composition, and sensory quality (Tonutti *et al*., 2023). The early stage (S1) is characterized by rapid growth driven by intense cell division (Pavel and DeJong, 1993; Lombardo *et al*., 2011). During S2, endocarp lignification occurs via activation of the phenylpropanoid and lignin biosynthetic pathways, temporarily slowing mesocarp enlargement (Dardick and Callahan, 2014). This stage is reduced or absent in early-ripening cultivars. In S3, growth resumes through cell expansion, accompanied by the accumulation of sugars and organic acids (Etienne *et al*., 2002) until the transition to S4, when fruit reaches its final size and ripening initiates (Brummell *et al*., 2004). Auxin promotes the onset of S4 by enhancing ethylene biosynthesis (Trainotti *et al*., 2007), leading to a climacteric rise in respiration and autocatalytic ethylene production mediated by the ACS1–ACO1 pathway, which triggers texture changes, colour development, and flavour formation (Rasori *et al*., 2002; Tatsuki *et al*., 2013).

Fruit maturity date (MD) is a key trait in peach, directly affecting orchard management, harvest scheduling, post-harvest handling, and fruit quality (Crisosto *et al*., 1997). Peach cultivars exhibit wide variability, ranging from very early (May) to ultra-late (October) maturity in the Northern hemisphere (Baccichet *et al*., 2025). Although MD has a strong genetic basis, it is also shaped by environmental and agronomic factors, including temperature regimes affecting the accumulation of growing degree hours (Souza *et al*., 2019), as well as crop load, water availability, and canopy microclimate (Yamaguchi *et al*., 2002; Reighard *et al*., 2017).

Because of its agronomic relevance, MD has been the target of numerous genetic studies, all reporting high heritability and a predominant genetic component. Early mapping work identified a major-effect quantitative trait locus (QTL) on linkage group 4 (LG4; *qMD4.1*), with pleiotropic effects on ripening date and quality traits (Dirlewanger *et al*., 1999; Eduardo *et al*., 2011). The presence and stability of this locus have been confirmed across multiple peach families, where it explains a large fraction of phenotypic variation, often with discrete bimodal or trimodal distributions (Bassi *et al*., 1988; Fresnedo-Ramírez *et al*., 2015; Rawandoozi *et al*., 2021). Fine mapping of the locus narrowed the interval to an ∼220 kb genomic region, containing two NAC transcription factor genes: *Prupe.4G187100* (ppa007577m, *NAC1*), highly similar to the tomato *NON-RIPENING* (*NOR*) gene, and *Prupe.4G186800* (ppa008301m, *NAC5*), closely related to *ANAC072/055/019*, a gene primarily involved in stress responses (Pirona *et al*., 2013). A 9 bp INDEL in the third exon of *NAC5* (alleles *NAC_9_* and *NAC_0_*) was proposed as the causal variant for *qMD4.1*. Despite the predictive value of this variant across different germplasm, a substantial portion of phenotypic variability remained unexplained (Cao *et al*., 2023; da Silva Linge *et al*., 2024), suggesting the presence of additional allelic diversity. A large deletion encompassing the entire *NAC5* gene up to the proximal *NAC1* promoter [∼500 bp from the transcription start site (TSS)] was also mapped in the same genomic region and underlies the recessive slow-ripening (*sr*) phenotype, in which fruits fail to ripen normally (Eduardo *et al*., 2015; Nuñez-Lillo *et al*., 2015). Functional evidence increasingly pointed to *NAC1* as the primary regulator of ripening, as it showed fruit-specific expression and a climacteric-associated transcriptional peak (Cao *et al*., 2023). Nevertheless, no sequence variants in *NAC1* regulatory or coding regions have been conclusively associated with MD, leaving the molecular basis of *qMD4.1* unresolved.

Insights into the genetic regulation of fruit ripening have largely originated from tomato, where natural mutants identified key transcriptional regulators such as NOR, CNR, and RIN, belonging to the NAC, MADS-box, and SBP-box families, respectively (Vrebalov *et al*., 2002; Giovannoni *et al*., 2004; Manning *et al*., 2006). Among these, NOR functions as a master regulator coordinating ethylene biosynthesis, cell wall modification, pigment accumulation, and aroma formation during ripening (Osorio *et al*., 2011). The central role of NAC transcription factors in ripening regulation appears conserved across species, as demonstrated for example in apple (*MdNAC18.1*), strawberry, banana, and kiwifruit (Martín-Pizarro *et al*., 2021; Li *et al*., 2024; Yue *et al*., 2024; Wu *et al*., 2025).

In this study, we dissect the genetic architecture of maturity date in peach and resolve the long-standing ambiguity surrounding the *qMD4.1* locus. We demonstrate that the previously proposed INDEL does not explain MD variation. Instead, we identify a multi-allelic series of structural variants in the upstream regulatory region of *NAC1* (named *Md*) that perfectly co-segregate with ripening time across different mapping families and germplasm. Beyond establishing the causal variants, this work provides robust diagnostic markers enabling accurate allele-based prediction for marker-assisted breeding.

## Materials and methods

### Plant materials and phenotyping

Accessions, breeding selections, and 151 F_1_ progenies from ‘Dulcebo66’×‘Pulchra’ belong to the University of Milan collection located in the experimental farm ‘M. Neri’ of RiNOVA in Imola (Emilia-Romagna region, Italy). Seedlings were own-rooted and planted at a distance of 1×4 m (within and between rows, respectively), trained as free central leader, and managed according to standard cultural practices for irrigation, fertilization, and pruning. Accessions and breeding selections were grafted on GF677 rootstock, trained according to the open vase system, and regularly spaced at a distance of 4×2.5 m. Fruits were thinned within 40–60 d after bloom, setting a crop load proportional to tree vigour. The MD trait was recorded when 2–3% of the fruits on the tree had reached full physiological maturity, assessed by sensory and visual inspection, also recording the IAD index (DA-meter, TR-Turoni, Forlì, Italy) and fruit firmness (puncture test by a digital penetrometer). MD was expressed as the number of days from 1 January (Julian days, JD). MD was evaluated for 2 years (2014 and 2016). Additional segregating populations previously used for MD QTL mapping included Bt×Ak (*n*=72) and Bt×Nr (*n*=40) (Serra *et al*., 2017); F_2_ E×E (*n*=72) (Kalluri *et al*., 2022); F_2_ W×By (*n*=102) and F_2_ C×A (*n*=302) (Pirona *et al*., 2013); and M×R (*n*=69) (Ciacciulli *et al*., 2018). Detailed phenotypic data and haplotypes at the *Md* locus are provided in Supplementary Table S1.

### Genotyping, linkage map construction, and quantitative trait locus mapping

The 18 K peach single nucleotide polymorphism (SNP) array was used to genotype the D×P population and the accessions panel, as previously described (Cirilli *et al*., 2021). Genotyping data were initially filtered for marker missing rate <10%. Non-informative markers, including monomorphic or non-segregating SNPs, as well as redundant loci with an identical segregation pattern (genotype similarity >0.95), were excluded. Markers showing distorted segregation from the expected 1:1 ratio for pseudo-testcross progeny were removed using χ^2^ goodness-of-fit tests (*P<*0.05). A total of 2033 and 2826 informative markers were retained for D66 and P, whose physical positions were retrieved from the peach reference genome assembly V2.0. Single-marker analysis was performed in Tassel 5.2.15 (Bradbury *et al*., 2007) using a general linear model (GLM), testing marker genotype as a fixed effect. D×P linkage maps were constructed following the two-way pseudo-testcross strategy for outcrossing species using JoinMap v4.1 (Van Ooijen, 2006), coding SNPs as lm×ll and nn×np configurations, respectively, for the seed and pollen parents. Linkage groups were defined using a minimum logarithm of odds (LOD) value of 10.0 using the regression algorithm (Kosambi mapping function), with a recombination frequency threshold of 0.4, LOD value of 1.0, and a goodness-of-fit jump of 5.0. The MapQTL v6.0 software was used for detecting QTLs. Significance thresholds were calculated by the random permutation test with 10 000 replicates considering the genome-wide LOD scores corresponding to *P*=0.05. The interval mapping function was employed for QTL detection with 95% significance and estimation of the percentage of phenotypic variation. MapChart v2.1 software was used to draw the mapped QTLs and the LOD plots.

### DNA extraction and resequencing

For each sampled accession, fresh young tissue was collected during spring, immediately snap-frozen in liquid nitrogen, and stored at −80 °C. High-molecular-weight (HMW) genomic DNA was extracted following the protocol described by Kang *et al*. (2023) with optimizations tailored for Oxford Nanopore Technologies (ONT) applications. Library preparation was performed using the Ligation Sequencing Kit v14 (SQK-LSK114, ONT). Each DNA sample meeting the quality requirements underwent a modified ligation-based library preparation workflow. A minimum input of 1.4 µg of HMW genomic DNA was used for the end-preparation step. Incubation times at 20 °C and 65 °C were extended to 30 min. Magnetic bead volumes were adjusted for each sample based on DNA concentration and initial fragment size distribution to maximize recovery of long fragments. Ethanol washing steps were repeated for samples showing lower purity. After the quality check step, DNA concentration and fragment size distribution were reassessed to determine whether to proceed with adapter ligation and clean-up. For adapter ligation, incubation with T4 DNA ligase and ligation adapters was extended to 30 min. Bead and washing buffer volumes were further adjusted to improve long-fragment retention. Different proportions of Long Fragment Buffer and Short Fragment Buffer were used during bead washing depending on fragment size profiles. The final quantification step was used to assess DNA concentration and estimate library molarity. A loading range of 10–20 fmol of DNA was considered suitable for sequencing. Molarity was calculated from DNA mass assuming a standard average molecular weight per base pair. Each sample was evaluated considering its initial electrophoretic profile. A MinION Mk1C device (ONT), running MiniKNOW software, was used for sequencing. In parallel, whole-genome sequencing (WGS) was performed for selected accessions using Illumina short-read sequencing to support variant discovery and genotyping analyses.

### Genomics analyses

Genomic analyses were performed using both long- and short-read sequencing approaches. For ONT long-read libraries, raw signals from the MinION device were basecalled using Dorado, and FASTQ reads were assessed for quality, read-length distribution, and total yield using NanoPack. Reads were filtered to retain those with a minimum Q-score of 8 and a minimum read length of 1000 bp, and mapped to the *P. persica* ‘Lovell’ reference genome v2.0 (Verde *et al*., 2017) using minimap2 (Li, 2018). For Illumina short-read libraries, reads were quality-filtered and mapped to the same reference genome using the bwa-mem algorithm. Variant calling was performed using FreeBayes v1.3.9, and downstream file handling and filtering steps were carried out using samtools, bcftools, and vcftools. All analyses were run using Python v3.7.

### Validation and genotyping of *Md* alleles

Genomic DNAs were extracted from young leaves by the cetyltrimethylammonium bromide (CTAB) protocol. Several primer pairs were designed and tested to genotype each *Md* allele. The final primer sets and annealing temperatures are indicated in Supplementary Table S1. PCRs were carried out using Go-Taq™ in a volume of 20 μl, containing 20 ng of peach genomic DNA. PCR was carried out under the following conditions: *Md_WT_* (WT) and *Md_DP_* (DP): 2 min at 95 °C, 30 cycles of 30 s at 95 °C, 30 s at 60 °C, and 90 s at 72 °C, followed by a 5 min final extension at 72 °C; *Md_M_/Md_WT_* (M/WT): 2 min at 95 °C, 30 cycles of 30 s at 95 °C, 3 min at 60 °C, and 90 s at 72 °C, followed by a 15 min final extension at 72 °C; *Md_DP3_* (DP3): 2 min at 95 °C, 30 cycles of 30 s at 95 °C, 1 min at 60 °C, and 90 s at 72 °C, followed by a 5 min final extension at 72 °C. PCR products were examined using agarose gel electrophoresis. Primer sets were applied sequentially in the following order: WT, DP, M/WT, and DP3.

### Fruit sampling and RNA sequencing analyses

To characterize NAC1 transcriptional dynamics during fruit development, an RNA-seq time course was generated using the early- and late-ripening cultivars ‘Springcrest’ and ‘O’Henry’. Fruits were collected at 13, 25, 36, 57, 71, 84, and 96 days after full bloom (DAFB) and at 16, 28, 39, 50, 64, 85, 106, 143, and 157 DAFB, respectively, during the 2023 growing season. All time points were represented by two or three biological replicates (Supplementary Table S1). Mesocarp tissue was immediately dissected, flash-frozen in liquid nitrogen, and stored at −80 °C prior to RNA extraction. Total RNA was extracted using a CTAB–LiCl protocol and treated with DNase I to remove genomic DNA contamination. RNA-seq libraries were prepared using the Illumina TruSeq mRNA protocol. Raw reads were trimmed with TrimGalore (adapter and quality trimming). Transcript abundance was quantified using Salmon (v1) (Patro *et al*., 2017) in quasi-mapping mode, indexing the *P. persica* v2.0 transcriptome with a k-mer size of 31. Expression values were imported into R using tximport and summarized to gene level as transcripts per million (TPM). Public RNA-seq datasets from additional peach genotypes were also retrieved and processed following the same pipeline (Supplementary Table S1). These datasets include annotations of sampling period (DAFB) and developmental stages as reported in the original studies, and were used as external validation to assess the generality of observed expression patterns across different *Md* allelic backgrounds. Because datasets differ in sampling schemes and growth conditions, comparative analyses focused on the relative timing of transcriptional activation rather than absolute transcript abundance, allowing a qualitative interpretation of expression dynamics across *Md* genotypes.

### Epigenomic datasets and data processing

Public epigenomic datasets used in this study are listed in Supplementary Table S1 and include ChIP sequencing (ChIP-seq) and DNA affinity purification sequencing (DAP-seq) experiments targeting the peach transcription factor NAC1, histone modifications (H3K4me3, H3K27ac, and H3K27me3), DNase I hypersensitivity assays, and whole-genome bisulfite sequencing (WGBS) data. All datasets were derived from the fruit ENCODE project (Lü *et al*., 2018), except for DAP-seq (Cao *et al*., 2023), and were generated from peach mesocarp tissues. Although cultivar background was heterogeneous, developmental stages are consistently annotated according to peach fruit developmental stages: ChIP-seq and DAP-seq were sampled at the S4 stage, and H3K4me3, H3K27ac, H3K27me3, DNase I hypersensitivity, and WGBS at S1 and S4 stages. Epigenomic signals were analysed in a locus-centred manner, focusing on the genomic region upstream of NAC1 on chromosome 4 (Pp04: 11 118 000–11 145 000 bp). This interval encompasses the entire upstream intergenic region extending from the annotated NAC1 TSS to the neighbouring gene boundary. Chromatin features were examined in their genomic context by assessing spatial co-occurrence patterns between NAC1 binding, chromatin accessibility, histone modification profiles, and DNA methylation across developmental stages. When available, biological or technical replicates were averaged, and profiles were summarized by grouping samples into immature (S1) and mature classes (S4). Raw sequencing reads were uniformly processed using standardized pipelines. Adapter sequences and low-quality bases were removed using Trim Galore (v0.6.10), and quality-filtered reads were aligned to the peach reference genome ‘Lovell’ v2.0. For ChIP-seq, DAP-seq, DNase-seq, and histone modifications (H3K4me3, H3K27ac, and H3K27me3), reads were aligned using minimap2. Signals were summarized as per-base read coverage across the upstream regulatory interval of NAC1 on chromosome 4 (Pp04: 11 118 000–11 145 000 bp). Coverage profiles were computed using pileup-based counting, smoothed using a Gaussian filter (σ=20–50 bp depending on dataset), and averaged across biological replicates within each assay and developmental stage. For visualization, signals were locally normalized within each chromatin mark to emphasize relative enrichment patterns along the locus. For WGBS, reads were aligned using Bismark (Krueger *et al*., 2011) with Bowtie2 backend (Langmead and Salzberg, 2012). Cytosine methylation levels were extracted for CG, CHG, and CHH contexts and quantified as the fraction of methylated reads over total reads at each cytosine position, yielding values ranging from 0 (fully unmethylated) to 1 (fully methylated). Custom scripts used for epigenomic data processing and visualization are available in the GitHub repository (https://github.com/marcoc83/peachMD).

## Results

### Maturity date mapping in D×P progeny

**Fig. 1.**
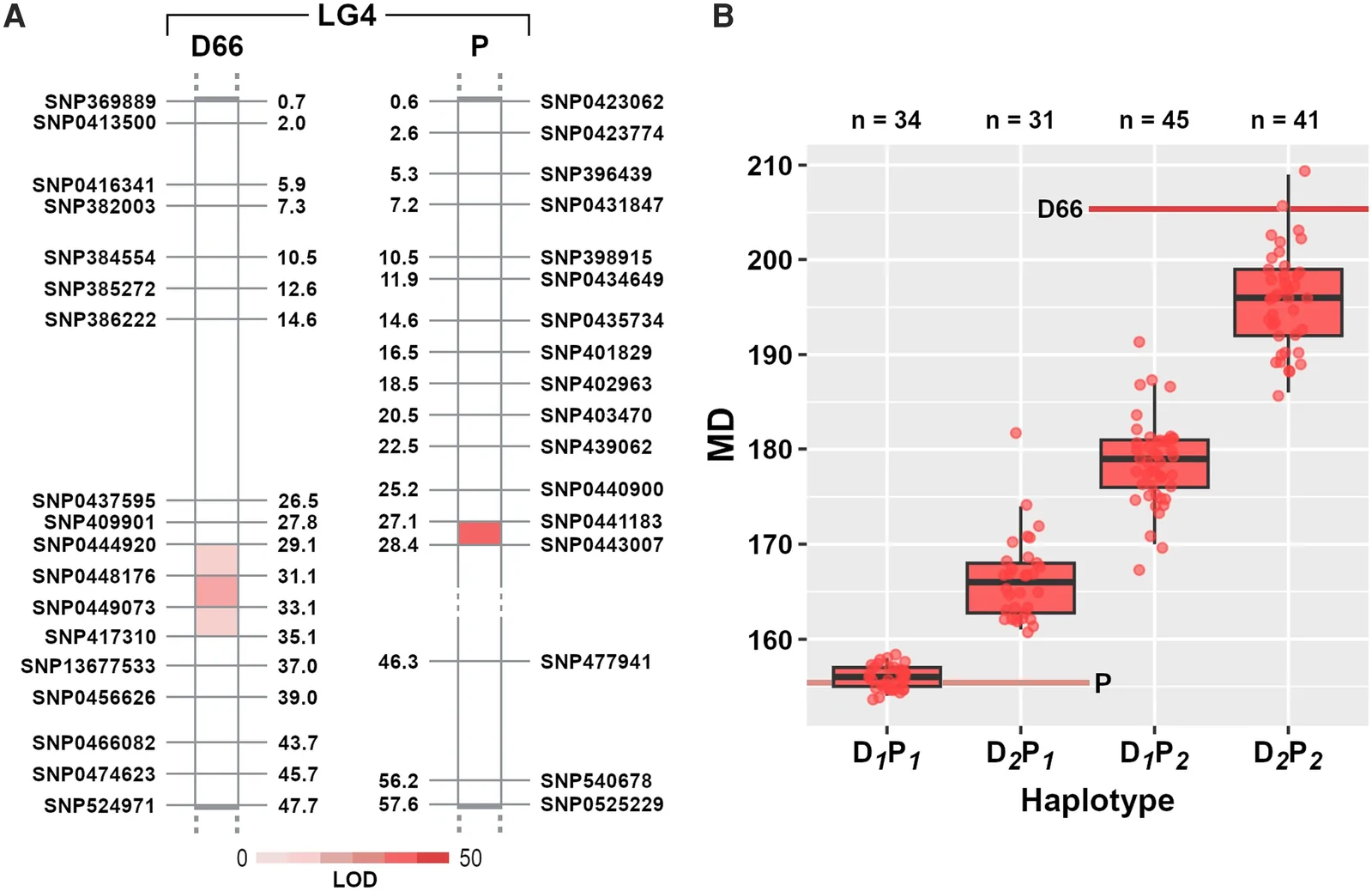
QTL mapping for maturity date (MD) in the F_1_ D×P population. (A) Linkage maps of LG4 for ‘Dulcebo66’ (D66) and ‘Pulchra’ (P) parents, showing SNP positions and QTL peaks detected by interval mapping: in P, the strongest signal corresponds to SNP_AO_0443007 (LOD=38.9, 10.15 Mb), while in D66 the peak maps at SNP_AO_0444920 (LOD=6.5, 11.9 Mb). Recombinant-defined candidate intervals are highlighted in red. (B) Effects of the four parental haplotype classes on MD, illustrating additive contributions from both parents; the *D_1_P_1_* haplotype is associated with earlier maturity. Boxplots display MD values recorded over 2 years, and differences among haplotypes are all significant according to ANOVA (*P*<0.01).

An F_1_ population of 151 seedlings derived from the cross ‘Dulcebo66’ (D66)×‘Pulchra’ (P) (hereafter referred to as D×P) was phenotyped for MD over 2 years. D66 is a mid-season peach selection (205 JD), while P is an ultra-early peach (155 JD). In D×P, MD segregated across a range of ∼50 d (from 150 to 205 JD) with a strong year-to-year correlation. A single-marker QTL analysis was performed separately in the two parents to test associations with MD variation. A major QTL for MD was detected in chromosome 4 in both parents, although not fully overlapping (Supplementary Fig. S1). Linkage maps of LG4 covered 47 cM in D66 and 57 cM in P (Supplementary Table S1). Interval mapping identified significant QTL peaks at SNP_AO_0443007 (LOD=38.9, position 10.15 Mb, *R*^2^=0.69) in P and at SNP_AO_0444920 (LOD=6.5, position 11.9 Mb, *R*^2^=0.21) in D66 (Fig. 1A). Haplotype analysis around the region revealed four allelic classes, indicating additive effects contributed by both parents, with the *D_1_P_1_* haplotype associated with earlier maturity (Fig. 1B; Supplementary Table S1). Recombinant individuals (#063 and #186 in P; #064 and #136 in D66) enabled narrowing of the candidate intervals. In D66, the QTL was confined to the 10.7–11.9 Mb region, while in P recombinant genotypes excluded the upstream region up to SNP_IGA_408654 (10.2 Mb). The downstream boundary in P could not be fully resolved due to a large homozygous block with no informative recombination.

This genomic region is already known to harbour a major QTL for MD (*qMD4.1*) and a candidate 9 bp INDEL in the *NAC5* gene, associated with earlier maturity (Pirona *et al*., 2013). Hereafter, the 9 bp INDEL and the reference alleles were denoted as *NAC_9_* and *NAC_0_*, respectively. D66 was heterozygous *NAC_9_*/*NAC_0_* and P homozygous *NAC_9_*. In the D×P progeny, the NAC INDEL only co-segregated with D66-derived haplotypes *D_1_* and *D_2_*, indicating that a biallelic model cannot account for the observed segregation pattern and suggesting the presence of additional functional variation at *qMD4.1*, or of a tightly linked QTL contributed by ‘Pulchra’.

### Genomic variation at the *Md* locus

**Fig. 2.**
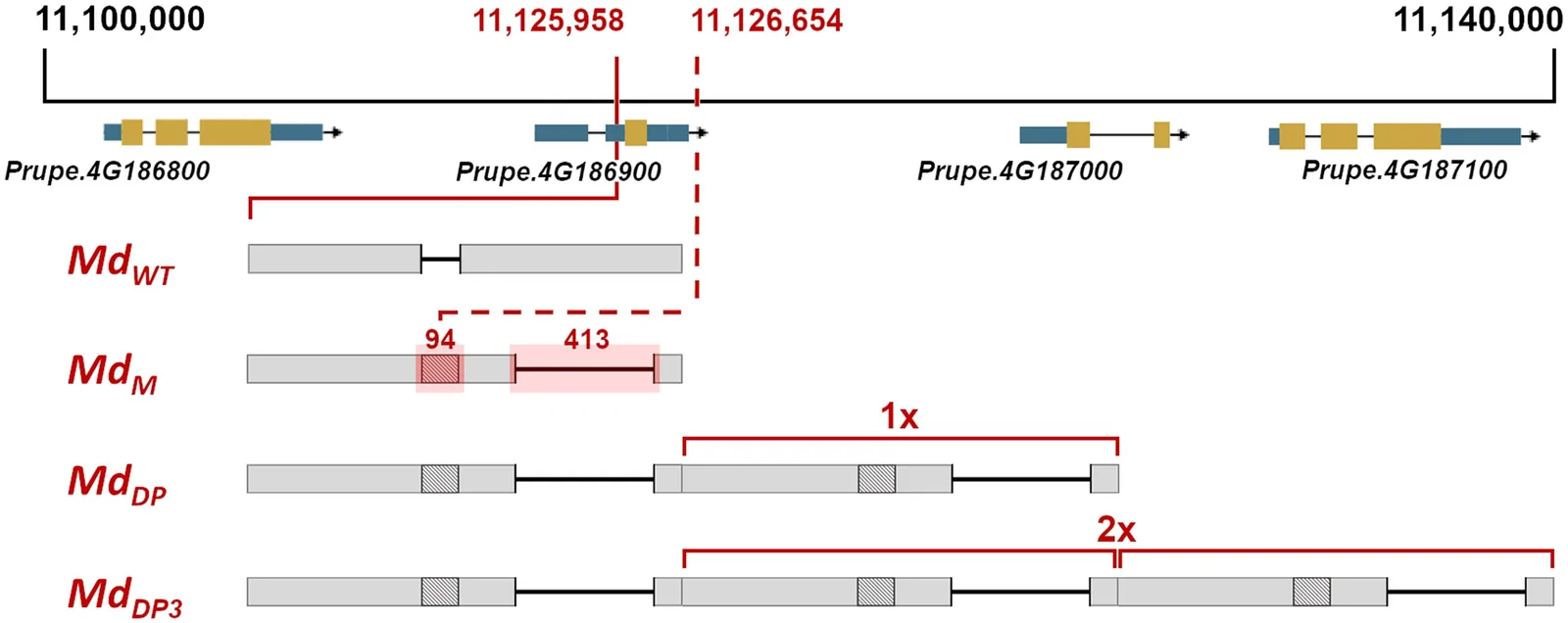
Structural variation at the *Md* locus. Four allelic variants were identified in the region between *Prupe.4G186800* (*NAC5*) and *Prupe.4G187100* (*NAC1*) and near the 3' UTR of *Prupe.4G186900*, a predicted long non-coding RNA. The reference-like allele (*Md_WT_*) corresponds to the structure present in the ‘Lovell’ reference genome. The *Md_M_* variant contains a composite rearrangement consisting of a 94 bp upstream tandem duplication insertion and a 413 bp downstream deletion (highlighted by the pink boxes) starting at ∼11 126 654 bp (red dashed line). The early-ripening alleles *Md_DP_* and *Md_DP3_* consist of a full duplication or triplication of the *Md_M_* structural unit, respectively, beginning upstream of *Md_M_* rearrangement, at 11 125 958 bp, resulting in two or three consecutive ∼1.5 kb copies (red continuous line). Grey boxes represent homologous blocks, hatched areas indicate the duplicated segment, and black bars mark deletion boundaries.

Genomic variations within the mapped region were characterized using 7.25 Gb of Illumina short reads and 5.74 Gb of ONT long reads from ‘Pulchra’, which was heterozygous for the putative causal mutation (assembly statistics in Supplementary Table S1). The combination of short- and long-read sequencing provided base-pair-level resolution and confirmed the presence of a largely homozygous region extending from 10.27 Mb to ∼17.0 Mb and several heterozygous SNPs and INDEL variants within the upstream 70 kb interval (10.21 Mb to 10.27 Mb). Interestingly, annotation revealed a single heterozygous locus within the extended homozygous block, harbouring two complex structural variants (SVs) compared with the ‘Lovell’ v2 reference genome (Supplementary Fig. S2). Both SVs mapped to approximately the same genomic region between the two NACs, encompassing *Prupe.4G186900*, a predicted long non-coding RNA gene. We referred to this locus as *Md*, assigning specific abbreviations to distinguish the different SVs. The first SV, named *Md_M_*, comprises a composite rearrangement occurring at ∼11 126 654 [near the 3'-untranslated region (UTR) of *Prupe.4G186900*] and consisting of an upstream 94 bp tandem duplication and a downstream 413 bp deletion compared with the ‘Lovell’ genome reference (named *Md_WT_*) (Fig. 2; Supplementary Fig. S3). The second SV, named *Md_DP3_*, corresponds to a tandem triplication of the *Md_M_* structural unit, beginning upstream of *Md_M_* rearrangement, at ∼11 125 958 bp (including part of the 5' UTR of *Prupe.4G186900*), resulting in three consecutive ∼1.5 kb copies (Fig. 2).

The presence of these SVs was further explored in other accessions sequenced with combined short- and long-read approaches. ‘Lucrezia’, an ultra-early season selection (∼145 JD) derived from self-pollination of ‘Maycrest’ (one of the two parents of ‘Pulchra’), was homozygous *Md_DP3_*; ‘Springcrest’, an early cultivar (∼170 JD) from which ‘Maycrest’ originated as a bud sport, carried an additional SV consisting of a simple duplication of the *Md_M_* unit, and named *Md_DP_* (Fig. 2), being heterozygous *Md_DP_*/*Md_WT_*. Two early-ripening nectarine cultivars, ‘Zhong You 4’ (ZY4) and its bud sport mutant ‘Li Xia Hong’ (LXH), which ripens 16 d earlier (Zhou *et al*., 2023), also carried *Md* SVs: ZY4 was heterozygous *Md_DP_*/*Md_WT_*, while LXH was homozygous *Md_DP_*. A PCR assay was developed to detect *Md* SVs (Supplementary Fig. S4) and used to genotype the D66 parent (which was heterozygous *Md_M_*/*Md_WT_*) and other early-ripening materials, including: ‘Mayfire’—one of the earliest ripening nectarines and parent of ZY4 and LXH (Li *et al*., 2013)—and its progenitor ‘Armking’, as well as ‘Springtime’, ‘Spring Baby’, and ‘Maycrest’: all were homozygous or heterozygous for *Md_DP_* except ‘Maycrest’, which carries *Md_DP3_* (Table 1).

**Table 1.** Summary of *Md* allelic composition across peach germplasm, breeding founder, and mapping parents

Accession	Origin	*Md*	MD (JD)	FDP (D)
Germplasm				
Lucrezia	Maycrest self	*Md_DP3_/Md_DP3_*	145	72
Spring Baby	Springcrest op×Springcrest op	*Md_DP_/Md_DP_*	153	80
Mayfire	Armking op	*Md_DP_/Md_DP_*	157	84
Springtime	(Lukens’s Honey×July Elberta)×Robin	*Md_DP_/Md_M_*	160	87
Maycrest	Springcrest mutation	*Md_DP3_/Md_WT_*	165	92
Springcrest	FV89-14×Springtime	*Md_DP_/Md_WT_*	170	97
Large White	Germplasm	*Md_M_/Md_M_*	178	110
Contender	Winblo×(Norman×NCA2679)	*Md_M_/Md_WT_*	215	140
Elegant Lady	Early O’Henry×July Lady	*Md_WT_/Md_WT_*	218	143
O’Henry	Merril Bonanza op	*Md_WT_/Md_WT_*	235	160
Zhong You 4	From Mayfire	*Md_DP_/Md_WT_*	*—*	79
Li Xia Hong	Zhong You 4 mutation	*Md_DP_/Md_DP_*	*—*	63
Redhaven	Halehaven×Kalhaven	*Md_M_/Md_WT_*	195	125
Cross-parents				
F_1_ C×A	Contender×Ambra	*Md_DP_/Md_WT_*	180	110
Rebus028	Big Top×Mayfire	*Md_DP_/Md_M_*	170	97
Max10	Unknown pedigree (Minguzzi)	*Md_WT_/sr*	255	180
BigTop	Unknown pedigree (Zaiger)	*Md_M_/Md_M_*	190	120
Armking	(Palomar×Springtime)×(Palomar×Springtime)	*Md_DP_/Md_M_*	174	105
Ambra	Stark Red Gold×Mayfire	*Md_DP_/Md_WT_*	180	110
Dulcebo066	sel.×(Contender×Elegant Lady)	*Md_M_/Md_WT_*	210	137
Pulchra	Vistarich×Maycrest	*Md_DP3_/Md_M_*	162	89
Nectaross	Stark Red Gold×Legrand	*Md_M_/sr*	221	148
Earligold	Complex from Robin	*Md_DP_/Md_M_*	165	92
Bounty	[(Halberta op)×Redskin]×(Loring×FV89-14) op	*Md_M_/Md_WT_*	203	130
NJ Weeping	Ornamental germplasm (PI)	*Md_WT_/Md_WT_*	235	160

Maturity date (MD) and fruit developmental period (FDP) are expressed in Julian days (JD) and days from blooming to maturity (D), respectively; *sr* indicates the slow-ripening allele.

The recurrent association between earliness and *Md* alleles across different early-ripening genetic backgrounds supports their involvement in determining maturity date.

### Association of *Md* alleles with maturity date across multiple segregating progenies

**Fig. 3.**
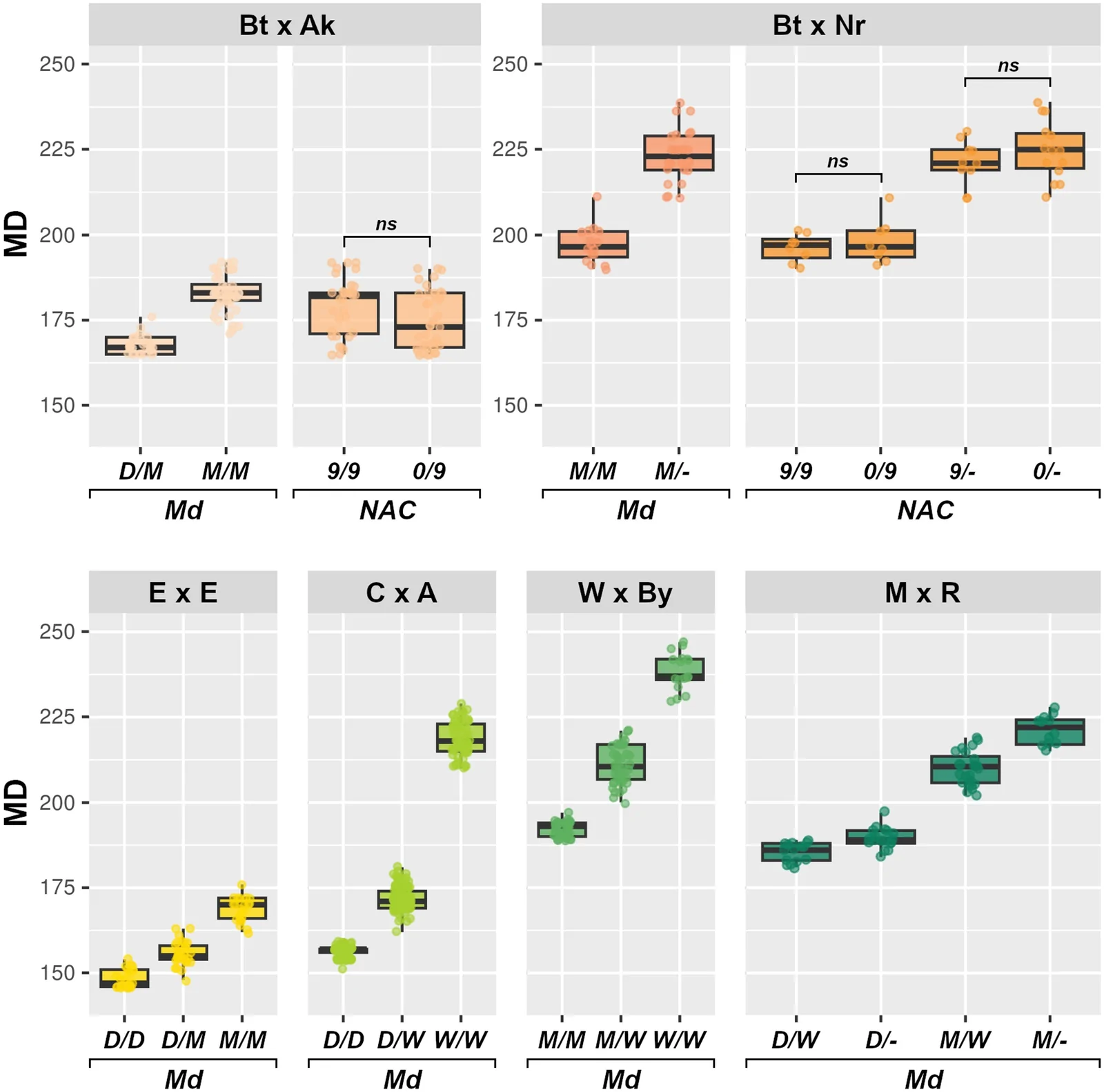
Association of *Md* alleles with maturity date (MD) across multiple segregating progenies. MD distributions are shown for *Md* and *NAC* genotypes in Bt×Ak, Bt×Nr, E×E, C×A, W×By, and M×R. Md alleles are coded as ‘D’ (*Md_DP_*), ‘M’ (*Md_M_*), ‘W’ (*Md_WT_*), and ‘–’ (*sr*, slow ripening allele). Means, SDs, and significance scores for each progeny are summarized in Table 2.

To provide an additional layer of validation for the association between *Md* alleles and MD, and to assess their relationship with the *NAC5* INDEL, we re-evaluated segregation patterns in several populations previously used for *qMD4.1* mapping. The entire progeny (when available) or a subset of individuals for each cross were genotyped to infer phase and co-segregation. Haplotypes, recombinant individuals, and statistical analyses across all populations are summarized in Supplementary Table S1. Segregation patterns across the different progenies are illustrated in Fig. 3 and Table 2.

**Table 2.** Summary of *Md* and *NAC* genotypic classes across multiple populations, their association with maturity date (MD), and significance levels

Pop	*Md–NAC* segregation	Genotype	No.	MD	*P*-value	*Md–NAC* linkage	Note
D×P	*M/WT×DP3/M*	*Md_DP3_/Md_M_*	33	155.7±1.1	*P*<0.001	D: *Md_M_*↔*NAC_9_* *Md_WT_*↔*NAC_0_* P: *Md_DP3_*↔*NAC_9_* *MdM*↔*NAC9*	Partial linkage
		*Md_DP3_/Md_WT_*	32	166.1±4.5			
		*Md_M_/Md_M_*	45	178.3±4.5			
		*Md_M_/Md_WT_*	41	195.5±5.1			
	*0/9×9/9*	*NAC_0_/NAC_9_*	73	182.6±15.4	*P*<0.001		
		*NAC_9_/NAC_9_*	78	168.7±11.7			
Bt×Ak	*M/M×DP/M*	*Md_DP_/Md_M_*	28	167.4±2.6	*P*<0.001	Bt: *Md_M_*↔*NAC_9_* *Md_M_*↔*NAC_0_* Ak: *Md_DP_*↔*NAC_9_* *Md_M_*↔*NAC_9_*	Segregation of Bt alternative *Md_M_—NAC_0_* haplotype
		*Md_M_/Md_M_*	44	182.6±5.5			
	*9/0×9/9*	*NAC_0_/NAC_9_*	33	178.9±8.5	*P*>0.039 ns		
		*NAC_9_/NAC_9_*	39	174.7±8.6			
Bt×Nr	*M/M×M/sr*	*Md_M_/Md_M_*	16	197.0±5.3	*P*<0.001	Bt: *Md_M_*↔*NAC_9_* *Md_M_*↔*NAC_0_* Nr: *Md_M_*↔*NAC_9_*	Segregation of Bt alternative *Md_M_—NAC_0_* haplotype Nr segregate for *sr* allele
		*Md_M_/sr*	24	223.2±7.8			
	*9/0×9/sr*	*NAC_0_/sr*	14	224.7±8.5	*P*>0.136 ns		
		*NAC_9_/sr*	11	220.1±6.9			
		*NAC_9_/NAC_0_*	7	196.1±4.2	*P*>0.993 ns		
		*NAC_9_/NAC_9_*	8	195.1±4.1			
F_2_ E×E	*DP/M×DP/M* *9/9×9/9*	*Md_DP_/Md_DP_*	25	148.3±2.3	*P*<0.001	*Md_DP_*↔*NAC_9_* *Md_M_*↔*NAC_9_*	No NAC segregation
		*Md_DP_/Md_M_*	26	155.6±3.1			
		*Md_M_/Md_M_*	21	168.8±3.2			
F_2_ W×By	*M/WT×M/WT* *9/0×9/0*	*Md_M_/Md_M_*	41	191.8±2.1	*P*<0.001	*Md_M_*↔*NAC_9_* *Md_WT_*↔*NAC_0_*	Perfect linkage
		*Md_M_/Md_WT_*	44	210.8±5.4			
		*Md_WT_/Md_WT_*	17	238.2±5.5			
F_2_ C×A	*DP/WT×DP/WT* *9/0×9/0*	*Md_DP_/Md_DP_*	94	156.2±1.4	*P*<0.001	*Md_DP_*↔*NAC_9_* *Md_WT_*↔*NAC_0_*	Perfect linkage
		*Md_DP_/Md_WT_*	143	171.4±3.2			
		*Md_WT_/Md_WT_*	65	218.5±4.8			
M×R	*WT/sr×DP/M* *0/sr×9/0*	*Md_DP_/sr*	18	189.4±2.7	*P*<0.001	M: *Md_WT_*↔*NAC_0_*	Partial linkage M segregate for *sr* R *Md_M_–NAC_0_* haplotype (not informative)
		*Md_M_/sr*	12	221.4±4.3			
		*Md_M_/Md_WT_*	24	209.8±4.9		R: *Md_DP_*↔*NAC_9_* *Md_M_*↔*NAC_0_*	
		*Md_DP_/Md_WT_*	15	185.3±2.7			

The table reports haplotype relationships between loci, highlighting cases of perfect and partial linkage, as well as non-informative progenies. Abbreviations: MD, maturity date; *n*, sample size; *sr*, slow-ripening allele; *NAC_0_* (0) and *NAC_9_* (9), for *NAC* allele; *Md_WT_* (WT), *Md_M_* (M), *Md_DP_* (DP), and *Md_DP3_* (DP3) for *Md* alleles; ns, not significant.

In the D×P population, according to the *Md*_WT_/*Md_M_×Md_DP3_*/*Md_M_* cross configuration, *Md_DP3_* was linked to the early-ripening *P_1_* haplotype, *Md_M_* to *P_2_* and *D_1_*, and *Md*_WT_ to the late-ripening *D_2_*, in perfect agreement with the four MD classes. However, the linkage of *NAC_9_–Md_M_* and *NAC_0_–Md_WT_* in the D66 parent does not allow determination of whether the observed effect corresponds to a single multi-allelic QTL (*Md*) or to two tightly linked QTLs (*Md* and *NAC*) (Table 2).

In ‘Big Top’×‘Armking’ (Bt×Ak) (Serra *et al*., 2017), the Ak parent was *Md_DP_*/*Md_M_* and homozygous *NAC_9_* whereas Bt was homozygous *Md_M_* and heterozygous *NAC_9_*/*NAC_0_*, an allelic configuration confirmed through ONT re-sequencing of Bt. Given the close proximity of *NAC* and *Md* (∼8 kbp), the alternative Bt haplotype *NAC_0_–Md_M_* provided a highly informative recombinant background for evaluating co-segregation between these loci. As in ‘Pulchra’, the Ak map was truncated at ∼10.5 Mbp due to a homozygous block of non-segregating SNPs extending to the end of the chromosome, with closest recombination occurring at ∼9.4 Mb. Consistent with the presence of a QTL only on LG4 of the Ak map, phasing of Ak-derived haplotypes confirmed the association of *Md_DP_* with the earlier-ripening class, whereas *NAC* alleles showed no significant effect (Table 2).

In ‘Big Top’×‘Nectaross’ (Bt×Nr), a QTL for MD was previously positioned on LG4 of the Nr map. The Nr parent was heterozygous for *Md_M_* and for the *sr* allele, a ∼26 kb deletion previously described (Eduardo *et al*., 2015). Segregation creates multiple configurations among *Md*, *sr*, and *NAC* alleles, but only *sr* and *Md_M_* clearly distinguished early- and late-ripening classes (*Md_M_*=198, *sr*=223 JD; *P*<0.01), without significant effect of NAC (Table 2). Thus, segregation was fully consistent with *Md* alleles while the recombination breakpoint observed in the ‘Big Top’ haplotype excludes a causal role for the *NAC5* INDEL variant.

We then re-analysed three F_2_ populations carrying a major MD QTL on LG4: ‘Contender’×‘Ambra’ (C×A), ‘Earlygold’ self-pollinated (E×E), and ‘NJ Weeping’×‘Bounty’ (W×By) (Pirona *et al*., 2013; Kalluri *et al*., 2022). The F_1_ C×A parent was heterozygous *Md_DP_*/*Md_WT_*, because it inherited the *Md_DP_* allele from ‘Mayfire’ (the male parent of ‘Ambra’), ‘Earlygold’ heterozygous *Md_DP_*/*Md_M_*, and the F_1_ W×By heterozygous *Md_M_*/*Md_WT_*, all in agreement with the observed segregations. In these progenies, the perfect linkage between *NAC_9_* and *Md_DP_* or *Md_M_* (and between *Md_WT_* and *NAC_0_*) probably masked the identification of the *Md* locus. Notably, the different SV combinations coherently match the distribution of ripening classes observed in the three progenies, from ultra-early individuals (homozygous *Md_DP_*, in E×E) to late ones (homozygous *Md_WT_* in C×A and W×By) (Fig. 3). PCR assays also identified several recombinant individuals in E×E (Supplementary Table S1). Moreover, heterozygous genotypes displayed partial dominance, particularly evident for *Md_DP_* over either *Md_M_* or *Md_WT_* in E×E and C×A, respectively and to a lesser extent for *Md_M_* over *Md_WT_* in W×By, with MD closer to the early-ripening class.

Finally, in F_1_ ‘Max10’×‘Rebus28’ (M×R) (Ciacciulli *et al*., 2018), M carried the *Md_WT_*/*sr* genotype while R was *Md_DP_*/*Md_M_*, a configuration fully consistent with the four observed MD classes (Fig. 3). However, R (originated from ‘Big Top’×‘Mayfire’) inherited the alternative *Md_M_* haplotype from Bt, which results in partial linkage between *Md* and *NAC* for three of the four allelic classes. This recombinant background explains why the *Md–NAC* association is maintained only for a subset of genotype combinations (as also reflected in the segregation patterns and linkage notes in Table 2), while being disrupted in others.

In summary, all mapped QTLs across the different populations share a common genomic segment encompassing the *Md* locus, whose multiple alleles consistently co-segregate with MD variation, while recombination events in some genetic backgrounds exclude a causal role for the 9 bp INDEL mutation.

### Association of *Md* alleles with maturity date in germplasm collections

To further substantiate the association observed across segregating populations, we realigned the assembled genomes of all parental lines to identify additional polymorphisms in linkage with *Md* alleles. By integrating QTL evidence from independent families with parental genome realignment and haplotype phasing, we were able to refine the *Md* locus and retain only variants truly compatible with the observed segregation patterns. Phase information from these haplotypes was also used to examine linkage patterns in other materials (cultivars and breeding selections) with known pedigrees, using founder haplotypes as reference.

Within the *Md_DP_* interval, fine-mapped to 10.88–12.10 Mbp in ‘F_1_ C×A’, the parents ‘Armking’, ‘Rebus28’, and ‘Earlygold’ were homozygous across the entire region except for the heterozygous *Md_DP_*/*Md_M_* allele. For *Md_DP3_*, an informative recombination in the ultra-early selection ‘Borgia’ (from selfing of ‘Maycrest’) delimited the downstream boundary at 11.3 Mb (Supplementary Table S1). Likewise, across this region, ‘Pulchra’ was homozygous except for the *Md_DP3_* allele. For *Md_M_*, the interval was relatively narrow (∼200 kb, from 10.98 Mbp to 11.20 Mbp in W×By) and contained (aside from *Md_M_*) only a limited number of variants consistent with segregation, including the *NAC5* INDEL, already excluded as causal (Supplementary Table S1). Collectively, genomic data reinforce the presence of a single multi-allelic causal locus at *qMD4.1*.

Given the abundance of publicly available short-read datasets, we tested a simplified approach to assess the detectability of *Md* SVs using short reads, which often fail to resolve complex structural rearrangements. In our case, standard alignment to the reference genome allows reliable detection of the *Md_M_* allele, due to the presence of ∼94 bp and ∼400 bp length polymorphisms relative to *Md_WT_*. However, this approach does not allow discrimination of *Md_DP_*, because tandem duplications do not generate an obvious loss of coverage or unique mapping pattern when reads are aligned to the reference sequence. To overcome this limitation, (i) the full-length *Md_WT_* sequence and (ii) the duplication junction were used as BLAST queries against short-read libraries. Using this targeted approach, *Md_M_*, *Md_WT_*, and *Md_DP_* could be reliably distinguished, even in heterozygosity (Supplementary Fig. S5). Nevertheless, *Md_DP_* could not be distinguished from *Md_DP3_* based on short reads alone, as they share an identical duplication junction (although the latter is present only in progenies derived from ‘Maycrest’). Detection of *sr*, instead, required whole-genome alignment, where it can be inferred from a local reduction in read coverage.

**Fig. 4.**
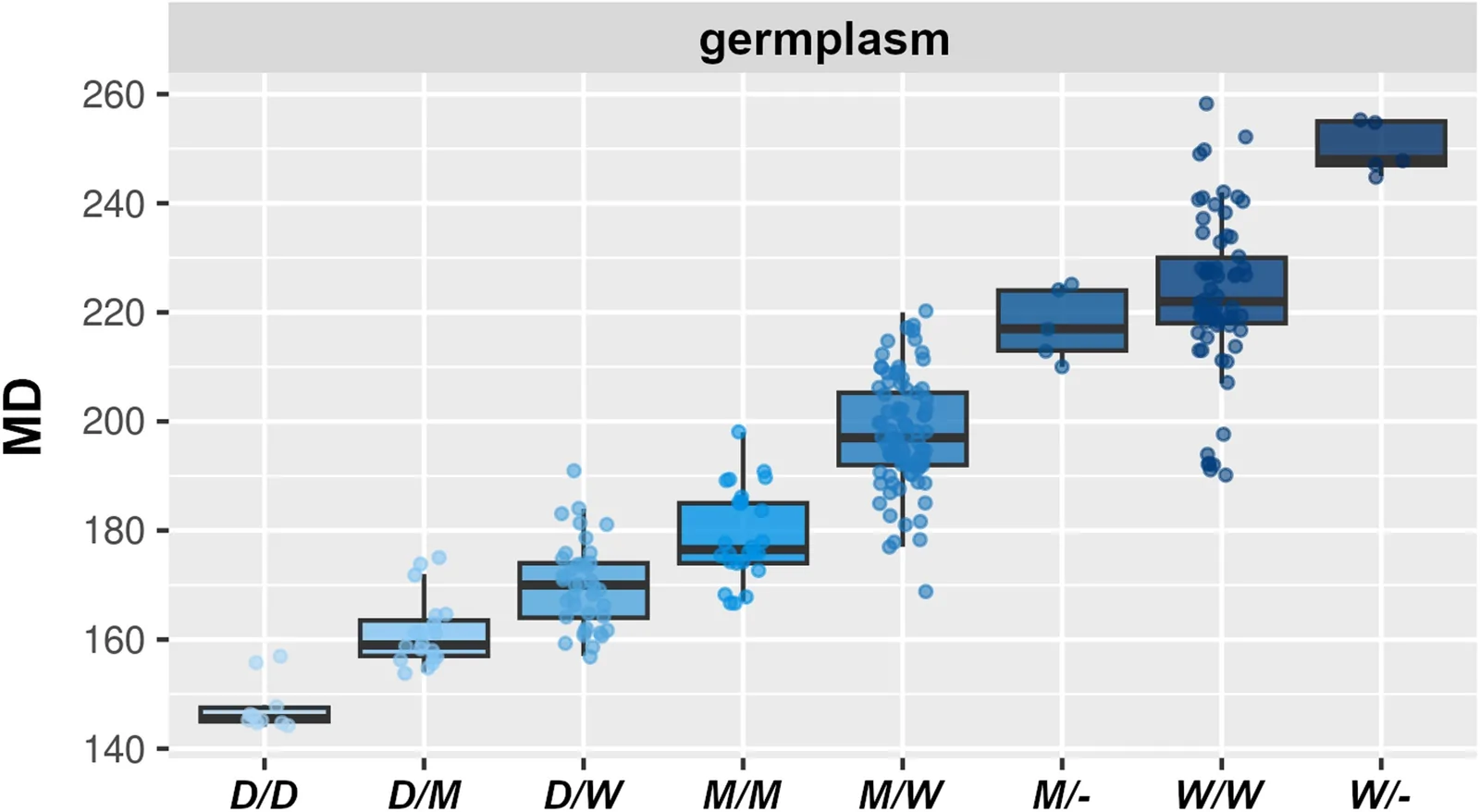
Boxplot of the association between *Md* alleles and maturity date (MD) in peach germplasm. *Md* genotypes stratify MD into distinct and additive classes across the germplasm panel, with all pairwise contrasts showing statistically significant differences (ANOVA, *P*<0.01). The observed phenotypic separation spans the full harvest window (∼140–250 JD). *Md* alleles are coded as ‘D’ (*Md_DP_*), ‘M’ (*Md_M_*), ‘W’ (*Md_WT_*), and ‘–’ (*sr*, slow ripening allele). Sample sizes per genotype class are as follows: M/W (*n*=81), W/W (*n*=73), D/W (*n*=38), M/M (*n*=30), D/M (*n*=21), D/D (*n*=10), W/− (*n*=5), and M/− (*n*=5).

Using publicly available WGS data and phenotypic records, we further assessed the association between *Md* alleles and MD in a broad set of accessions (Supplementary Table S1). Despite some residual variability, probably due to segregation of other QTLs, *Md* alleles clearly and additively separate maturity classes from ultra-early to late, collectively explaining nearly the full harvesting calendar in peach germplasm (140–250 JD) (Fig. 4). In predictive comparison, *Md* SVs [*R*^2^=75.4%, mean absolute error (MAE)=9.4] outperformed both the *NAC* (*R*^2^=48.6%, MAE=14.1) and the most strongly associated SNP on the peach array (*R*^2^=20.6%, MAE=19.06).

We also explored the entire *NAC1* upstream region in search of additional candidate polymorphisms. Aside from a previously reported 232 bp deletion located ∼500 bp upstream of the TSS, which does not co-segregate with MD (Pirona *et al*., 2013), no other variants showed patterns consistent with a functional role in maturity variation.

### Functional evidence of the effects of *Md* structural variants on *NAC1* expression

**Fig. 5.**
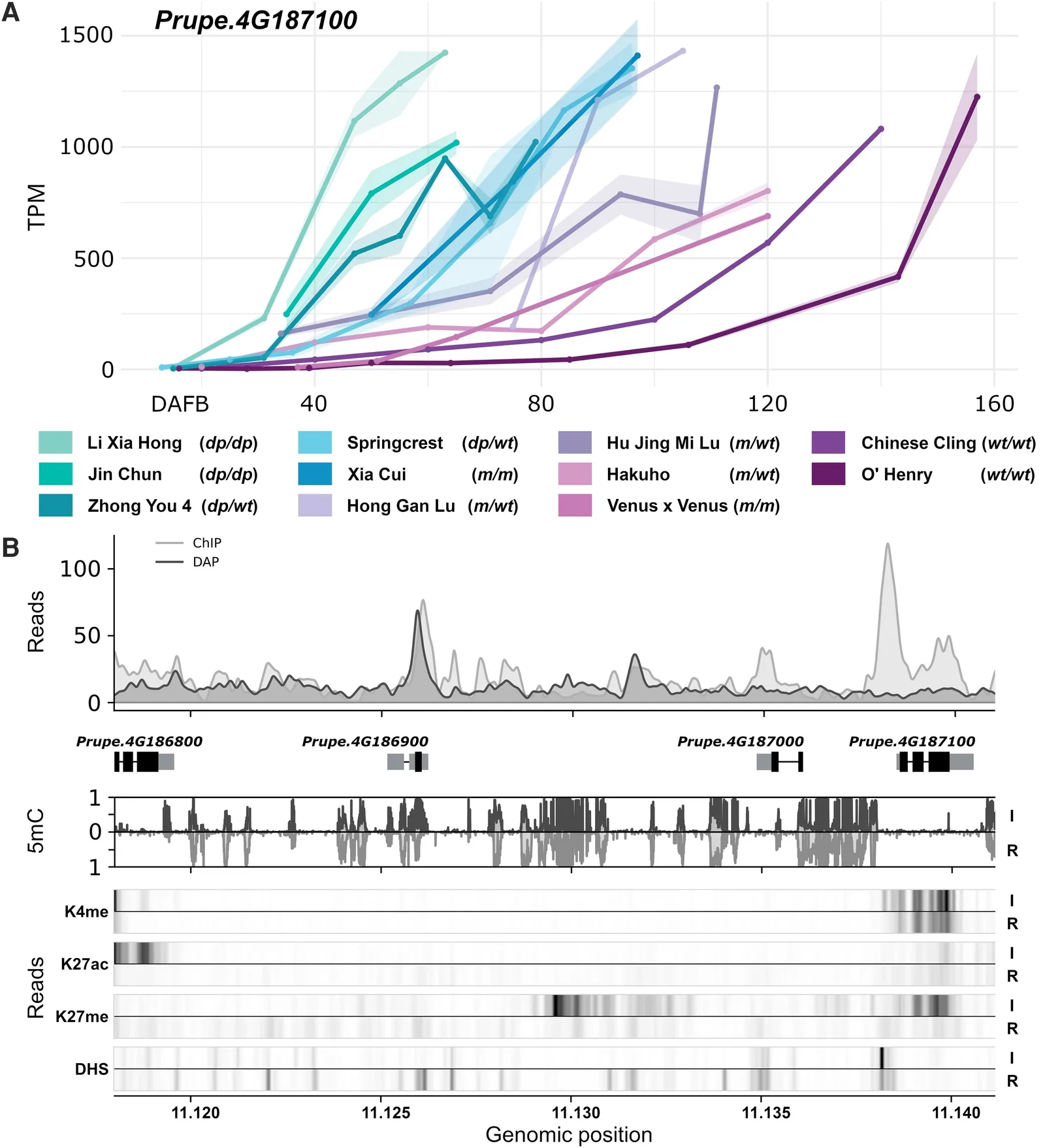
Functional evidence linking *Md* structural variants to *NAC1* expression dynamics and chromatin state. (A) Expression dynamics of *NAC1* (*Prupe.4G187100*) during fruit development in peach genotypes carrying distinct *Md* alleles. RNA-seq profiles are shown across developmental time, from early fruit development to ripening (days after full bloom, DAFB), as reported in the original studies. Curves represent the mean expression trend, with shaded areas indicating variability across available datasets. (B) Epigenomic profiles across the upstream regulatory region of NAC1. ChIP-seq and DAP-seq profiles highlight two regions of NAC1 binding: one proximal to the coding sequence and a second within the upstream regulatory region (∼11.126 Mb), coinciding with the insertion site of *Md* structural variants. DNA methylation (5mC) from fruit mesocarp, together with chromatin accessibility (DNase I hypersensitivity) and histone modification profiles (H3K4me3, H3K27ac, and H3K27me3), are shown for immature (I) and mature (R) fruit stages and indicate a chromatin environment permissive to transcription at this locus. Because datasets differ in sampling schemes, developmental staging, and growth conditions, both transcriptomic and epigenomic data are interpreted in terms of relative patterns and developmental timing.

To investigate the transcriptional dynamics of the *NAC1* gene, fruits of ‘Springcrest’ (SP) (*Md_DP_*/*Md_WT_*) and ‘O’Henry’ (*Md_WT_*/*Md_WT_*) were sampled at multiple developmental stages, from 20 DAFB until ripening, occurring at 96 and 157 DAFB, respectively. In both genotypes, *NAC1* transcript accumulation displayed an exponential trajectory culminating in a distinct peak at the onset of fruit ripening. However, the expression peak appeared earlier in SP, consistent with the presence of the *Md_DP_* allele (Fig. 5). To extend this observation, we analysed publicly available RNA-seq datasets for additional genotypes carrying distinct *Md* alleles (Supplementary Table S1). Those harbouring the *Md_DP_* allele (e.g. ‘LXH’, ‘ZY4’, and ‘Jin Chun’) exhibited an accelerated rise in *NAC1* expression, reaching the maturation-associated peak sooner than *Md_M_* or *Md_WT_* backgrounds. Although overall expression levels varied among genotypes, the timing of *NAC1* expression was consistently shifted according to allelic composition, indicating a dosage-dependent modulation of its transcription during fruit development. Conversely, *NAC5* expression was less clear, displaying strong genotype-dependent variability and no coherent temporal pattern, although transcript levels were generally higher at early developmental stages. *NAC1* transcription was markedly reduced in a slow-ripening genotype homozygous for the *sr* deletion, which spans *NAC5* and extends into the upstream regulatory region of *NAC1*, including the *Md* locus (Supplementary Table S1). In parallel, we noted that the adjacent gene *Prupe.4G186900*, probably encoding a long non-coding RNA (lncRNA), is transcriptionally active (albeit at low levels) predominantly in *Md_DP_* genotypes, including SP, LXH, ZY4, and JC. By contrast, this transcript is absent or detected only sporadically at late maturation stages in *Md_WT_* or *Md_M_* backgrounds, suggesting a possible link between allele-specific regulatory structure and lncRNA expression. Also, the other transcript annotated in the region (*Prupe.4G187000*) was barely detectable.

Publicly available epigenomic datasets were explored to obtain a qualitative view of the regulatory landscape at the *Md* locus during fruit development. Although derived from different genotypes and experimental contexts, these datasets collectively provide a useful framework for interpreting the chromatin landscape in this region and for identifying regulatory features associated with the *Md* locus. They include ChIP-seq and DAP-seq experiments targeting the transcription factor NAC1, profiles of histone modifications (H3K4me3, H3K27ac, and H3K27me3), DNase I hypersensitivity assays, and bisulfite sequencing, all generated from peach mesocarp tissue sampled at immature and mature fruit stages (Fig. 5; Supplementary Table S1). ChIP-seq data from ‘Babygold’ (*Md_M_*/*Md_WT_*) revealed two NAC1 regions of binding enrichment: one proximal to the coding sequence (∼11.138 Mb) and a second within the upstream regulatory regions (∼11.126 Mb), coinciding with the insertion site of the *Md* structural variants. Independent DAP-seq analysis in ‘Hu Jing Mi Lu’ (*Md_M_*/*Md_WT_*) confirmed a strong peak at the same upstream position, supporting the presence of a conserved regulatory interaction. Interestingly, the *Md_M_* rearrangement generates a duplication of the putative *NAC1*-binding motif ACG(T/C)(A/C) in this region, suggesting a potential modulation of autoregulatory interaction. Beyond NAC1 binding, chromatin features display hallmarks of regulatory activity, including DNase I hypersensitivity, enrichment of activating histone marks (H3K4me3 and H3K27ac), and a relative depletion of repressive mark H3K27me3. DNA methylation profiles show a locally structured pattern with alternating hypo- and hypermethylated segments, and a reproducible hypomethylated valley around the *Md* insertion site. No major qualitative differences were observed between immature and ripe fruit stages, indicating that this region maintains a permissive chromatin state throughout development.

### Origin and spread of *Md* alleles

**Fig. 6.**
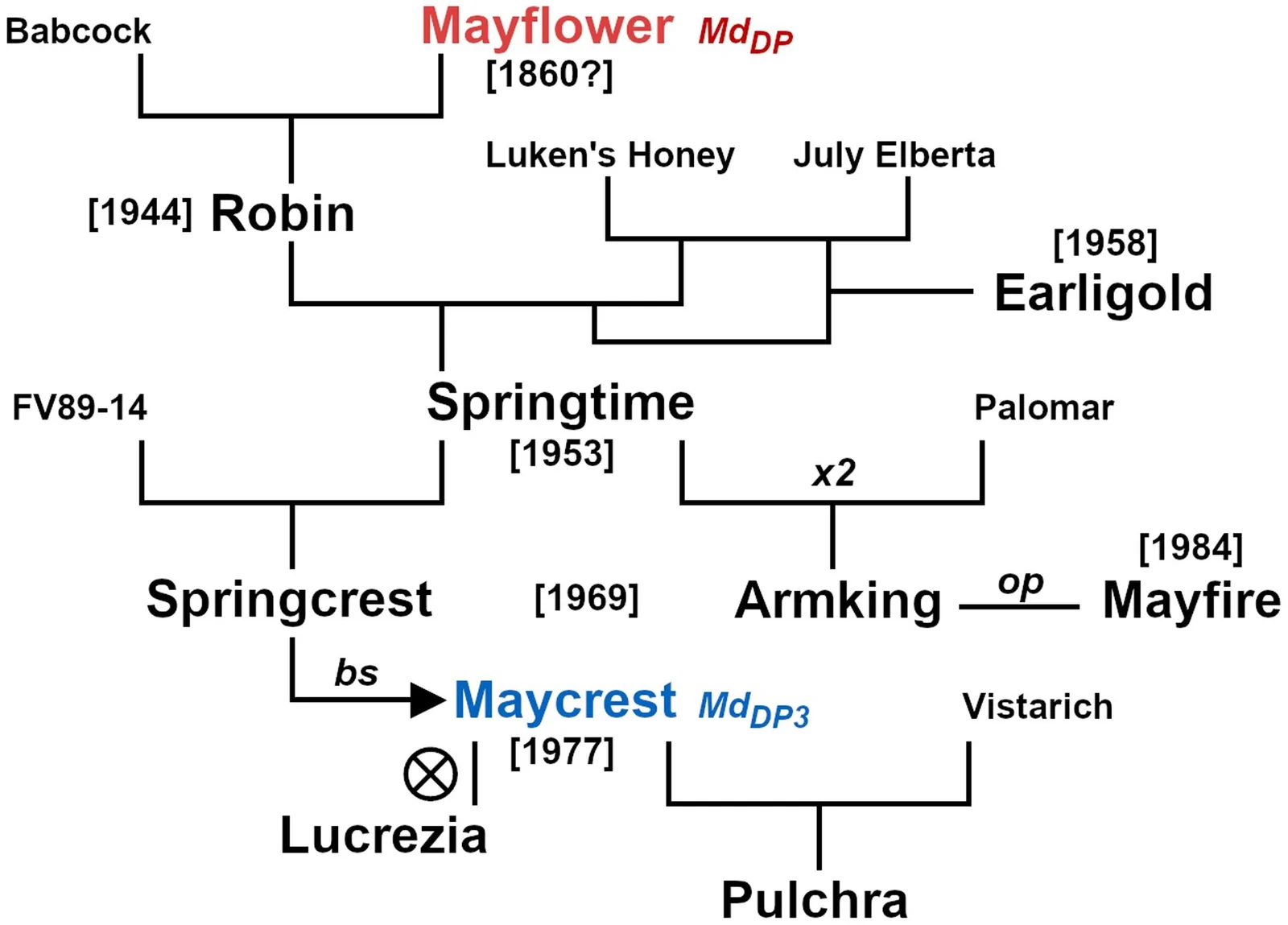
Pedigree of early-ripening peach cultivars tracing the origin of *Md_DP_* and *Md_DP3_* alleles. **‘**Mayflower’ introduces the early-ripening *Md_DP_* haplotype into modern germplasm through ‘Robin’ and ‘Springtime’. ‘Springcrest’ later gives rise to the bud sport ‘Maycrest’, in which an additional somatic duplication generates the *Md_DP3_* allele, transmitted to ‘Lucrezia’ and ‘Pulchra’. Years of release are shown in parentheses; bs=bud sport; op=open pollination; ×2 indicates repeated use of ‘Springtime’ as a parent.

In order to explore and trace the origin of the various *Md* SVs, genome sequences of wild peach relatives were investigated. The *Md_WT_* sequence is substantially conserved across species, except for several SNPs scattered throughout the region and a few INDELs, the most notable of which is ∼30 bp long and present only in peach, *P. kansuensis*, and apricot (Supplementary Fig. S6). Within peach, *Md_M_* is widespread in many Chinese landraces as well as in European and North American accessions. More intriguing is the case of the early-ripening *Md_DP_*, whose introduction traces back to the old cultivar ‘Mayflower’, of uncertain origin and probably identified around 1860 in North Carolina (Hedrick, 1917). Analyses of the ‘Mayflower’ accession (CREA-Rome collection) confirmed the presence of the *Md_DP_*, as well as in breeding materials initially developed by Armstrong Nurseries (Ontario, CA, USA) and University of Florence (Italy): ‘Robin’ (from Babcock×Mayflower), heterozygous *Md_DP_*/*Md_WT_*, and ‘Morettini1’ (from Cumberland×Mayflower, 1938) (Fig. 6). ‘Robin’ was the founder of important early varieties such as ‘Springtime’ (1953), ‘Earlygold’ (1958), and ‘Maytime’ (1958), followed later by the first early nectarine ‘Armking’ (1969) (Okie, 1998). In turn, ‘Springtime’ was the parent of ‘Springcrest’ (1969), but also of ‘Springold’ (1966), ‘Flordasun’ (1969), and ‘Royal Gold’, a bud sports isolated by C.F. Zaiger in 1967. According to pedigree information, ‘Maycrest’ originated as a bud sport of Springcrest discovered in 1977 and ripening 3–5 d earlier. Our genomic analyses show that only ‘Maycrest’ carries *Md_DP3_*, representing an additional copy-number expansion of the *Md_M_*-derived structural unit. This suggests that the bud sport event leading to ‘Maycrest’ probably involved a further duplication event at the *Md* locus. This is consistent with a somatic duplication event in the bud sport lineage. However, whether *Md_DP3_* systematically confers a measurable phenological shift relative to *Md_DP_* will require validation through controlled crosses and segregation analyses.

## Discussion

### Allelic determinants of maturity date in peach

The identification of causal variants underlying key agronomic traits is crucial to accelerate breeding progress in perennial fruit trees. In peach, MD is particularly important for defining the harvest calendar and market windows, and for its pleiotropic effects on internal and external fruit quality and post-harvest behaviour. Consequently, numerous studies have aimed to characterize the genetic architecture of MD, identifying major QTLs associated with early or late maturation in both biparental segregating populations and germplasm collections. The central region of chromosome 4 has repeatedly emerged as an MD hotspot due to the frequency with which QTLs have been mapped there and the large phenotypic variance they explain across diverse genetic backgrounds (Quilot *et al*., 2004; Dirlewanger *et al*., 2012; Romeu *et al*., 2014; Hernández Mora *et al*., 2017; da Silva Linge *et al*., 2021; Rawandoozi *et al*., 2021), segregating as a Mendelian trait in certain progenies such as C×A (Eduardo *et al*., 2011). A 9 bp INDEL located in the third exon of the *NAC5* gene was initially proposed as a candidate causal variant for MD in peach (Pirona *et al*., 2013). Association studies have consistently shown that this variant provides reasonable discriminatory power across different germplasm sets, though showing substantial phenotypic variability within genotypic classes (Cao *et al*., 2023).

In this study, we mapped a large-effect locus for MD to the central region of chromosome 4 in both parents of the D×P population. The segregation pattern could not be explained solely by the 9 bp INDEL, suggesting the presence of additional functional alleles at *qMD4.1* or a tightly linked QTL. The presence of a large fully homozygous region in the P parent (donor of the early-maturity allele) enabled the identification of a structural variant in the region (∼11.126 Mb) between *NAC5* and *NAC1* consistent with the observed segregation. By expanding the analysis to important breeding and germplasm materials, we identified a multi-allelic series at the *Md* locus, comprising the reference allele *Md_WT_* (‘Lovell’) and three structurally distinct derived alleles (*Md_M_*, *Md_DP_*, and *Md_DP3_*).

Reassessment of multiple segregating populations previously used for MD QTL mapping clearly showed a consistent co-segregation of *Md* allelic series with MD, predicting phenotypes from ultra-early to late ripening. The allelic effects were generally additive, with partial dominance particularly evident in the early alleles *Md_DP3_* and *Md_DP_*. Identification of an alternative haplotype in ‘Big Top’ allowed genetic decoupling of *Md* from the *NAC* variant, confirming the former as the causal locus at *qMD4.1*.

In addition to *Md* alleles, a series of large independent deletions (∼26 kb) encompassing the entire *NAC5* gene and most of the *NAC1* promoter have been associated with an *sr* trait when homozygous, and delayed maturation when heterozygous (Eduardo *et al*., 2015; Nuñez-Lillo *et al*., 2015). The *sr* phenotype is characterized by a block in the ripening process, resulting in fruits that fail to mature properly (Brecht *et al*., 1984). Recently, palindromic sequences flanking the deletion have been implicated in this phenomenon in a *de novo* somatic mutant (Pietrella *et al*., 2025). The *sr* allele helps to complement the *Md* allelic series and provides a mechanistic explanation for the full spectrum of phenotypic variation observed in segregating populations, accounting for late- or atypical-ripening classes in certain families (e.g. ‘Bt×Nr’ and ‘M×R’). We also assessed the association of *Md* alleles with MD by leveraging publicly available germplasm data, confirming their strong predictive power despite the likely segregation of additional QTLs.

Furthermore, we reconstructed the evolutionary and breeding history of *Md* alleles. The distribution of *Md_DP_* across germplasm and breeding materials appears recent and geographically limited compared with *Md_WT_* and *Md_M_*, which are widespread in traditional European, American, and Chinese germplasm. Comparisons with wild relatives indicate that the *Md_WT_* allele is ancestral and highly conserved (though with some polymorphisms) even in more distantly related *Prunus* species such as apricot. Pedigree analyses of major North American germplasm suggests that the early-ripening *Md_DP_* allele most probably entered modern breeding through the historical accession ‘Mayflower’—also known under synonyms such as ‘Fior di Maggio’ (Italy) and ‘Avant Pêche’ (France) (Okie, 1998). This accession is genetically closely related to other 19th century early-ripening cultivars, including ‘Amsden’ (selected by L.C. Amsden in Missouri, 1868) and ‘Ribet’ (Morettini and Bellini, 1971). Within this genealogical background, the emergence of *Md_DP3_* in ‘Maycrest’ represents a more recent copy-number expansion derived from the *Md_DP_* haplotype carried by ‘Springcrest’. Although *Md_DP3_* segregates with ultra-early ripening, whether it confers an effect distinct from *Md_DP_* under equivalent genetic backgrounds has not yet been formally demonstrated. The extensive block shared among several *Md_DP_*-bearing cultivars is consistent with the apparent monophyletic origin of this allele and reflects strong selection for earliness across decades of breeding. Such a combination of single-origin introgression and directional selection has resulted in a highly conserved haplotype surrounding the *Md_DP_* allele, which persists across multiple independent breeding lines.

### Functional evidence and transcriptional dynamics of the *Md* locus

Structural variation at the *Md* locus highlights a link between upstream regulatory architecture and NAC1 transcriptional control during fruit maturation. In particular, the structural rearrangements associated with *Md* alleles overlap an upstream region that is bound by NAC1 in both ChIP-seq and DAP-seq experiments. The co-occurrence of NAC1 binding with open chromatin, enrichment of activating histone marks, and local DNA hypomethylation indicates that this interval may represent an active regulatory region. These observations are compatible with a model in which NAC1 may engage in upstream autoregulatory interactions within a permissive chromatin environment, contributing to the fine-tuning of its transcript accumulation in an allele-dependent manner. Similar upstream autoregulatory architectures have been documented in apple for a NAC transcription factor (MdNAC18.1) (Zhang *et al*., 2025). Previous studies have examined the relative contributions of *NAC5* and the closely linked *NAC1* to peach fruit development and ripening. Based on their expression patterns, the two genes appear to perform distinct functions, displaying different tissue specificities and temporal dynamics. *NAC5* shows broad tissue expression, with the highest transcript abundance in flowers, roots, phloem, fruits, seeds, and leaves (Cao *et al*., 2023). In fruits, *NAC5* is predominantly expressed at immature stages, and its abundance decreases as ripening progresses. Moreover, when comparing expression profiles between mutant sports differing in ripening time and their donor genotypes, *NAC5* does not consistently show differential expression (Cao *et al*., 2023; Zhou *et al*., 2023). While *NAC5* is excluded as the causal determinant at *qMD4.1*, it may still contribute to other aspects of fruit development or ripening-related processes. In contrast, *NAC1* displays fruit-specific expression, peaking at the onset of climacteric ripening. Our results extend these observations by showing that the timing of the NAC1 expression peak is allele dependent: *Md_DP_* alleles are associated with an earlier *NAC1* peak, *Md_M_* alleles with intermediate timing, and *Md_WT_* alleles with later gene expression. In this context, the strong reduction of NAC1 transcription observed in the slow-ripening background may represent one of several genetic mechanisms contributing to the modulation of its expression.

Studies on NAC downstream genes in peach are limited, but yeast one-hybrid and dual-luciferase assays showed that NAC1 is able to interact with promoter sequences of key ripening genes, including those involved in ethylene biosynthesis (*ACS1* and *ACO1*), cell wall modification (*PG1*, *PME1*, and *PL1*), or aroma production (Jin *et al*., 2022; Cao *et al*., 2023; Zhang *et al*., 2024). NAC1 binding to NAC-motif-rich regulatory regions of ripening genes is consistent with a direct transcriptional activation mechanism (Cao *et al*., 2023). By contrast, NAC5 has far fewer validated targets. Recent evidence shows that NAC5/NAC1 tandem can activate *PGF*, a gene involved in peach softening, but its broader regulatory network remains unclear (Zhang *et al*., 2024).

Peach remains recalcitrant to genetic transformation, limiting species-specific functional validation. Heterologous complementation experiments in tomato have been widely used, although they provide contrasting evidence. Cao *et al*. (2023), using ‘Ailsa Craig’, reported no clear phenotypic changes in *NAC5*-overexpression lines (with or without the 9 bp insertion), whereas those overexpressing *NAC1* caused fruit to turn red ∼6 d earlier than the wild type. By contrast, Zhang *et al*. (2023) reported that overexpression of both genes accelerated ripening in the same tomato background, although *NAC1* produced a more pronounced effect. Overall, *NAC1* is emerging as a core regulator of the ripening network in peach, whereas *NAC5* appears to play an auxiliary role in developmental (or stress-related) processes.

Additional loci influencing fruit maturation in peach represent genetic mechanisms distinct from the *Md* locus but acting within ripening contexts in which NAC1 may be functionally implicated. A clear example is the *DBF* locus, which controls anthocyanin accumulation in blood-fleshed peaches, and is also associated with advanced ripening. Functional analyses have shown that the *DBF*-encoded NAC transcription factor (BL) can interact with NAC1 and promote the expression of ripening-related genes (Wang *et al*., 2024). These observations indicate that NAC1 may operate within a broader regulatory network in which distinct genetic factors converge on common transcriptional regulators to modulate the timing and progression of fruit ripening.

### Breeding implications and marker-assisted selection

We developed a set of diagnostic markers capable of discriminating the four structural variants at the *Md* locus. Together with existing *sr* markers, they provide a comprehensive and reliable genotyping toolkit for predicting MD in peach. Given the near-Mendelian effect of *Md* alleles (as clearly shown in Fig. 4 in the germplasm panel), this marker set enables accurate classification of genotypes into distinct ripening classes and provides a strong basis for marker-assisted selection. From an operational breeding perspective, the availability of these markers allows early prediction of maturity timing, facilitating rapid exclusion of undesired ripening classes and more efficient allocation of resources. By directly capturing the full allelic complexity of a major-effect locus, *Md*-targeted selection offers a robust and transferable alternative to SNP-based assays. At present, *Md* genotyping relies on PCR amplicon-based assays that are not readily scalable to high-throughput SNP platforms. Consistently, the SNP most strongly associated with *Md_DP/DP3_* (Peach_AO_0443007) showed limited predictive power, particularly in nectarines, and failed to recapitulate the multi-allelic structure of *Md* (Supplementary Fig. S7). Thus, while *Md*-targeted genotyping offers a practical and highly predictive marker-assisted selection tool, its widespread implementation will require routine use of specific primers and future development of more scalable assays capable of capturing the full allelic complexity of this locus.

### Conclusion

Our results identify *Md* as the major causal locus underlying *qMD4.1* in peach, with a multi-allelic series of SVs in the upstream regulatory region that appear to influence the timing and amplitude of *NAC1* transcription. From a breeding standpoint, *Md* genotyping provides a powerful predictor of MD, far outperforming either *NAC5* INDEL- or SNP-based assays that cannot capture the underlying allelic complexity. The diagnostic primer set developed provides a practical tool for marker-assisted selection and design of tailored harvest windows. Although genetic, structural, and transcriptional evidence converges on *Md*, definitive functional validation will require allele-swap or regulatory-editing experiments (e.g. introducing the *Md_DP_* upstream regulatory region into the wild-type background, and reciprocally, via CRISPR-mediated promoter replacement). Such assays would directly test whether the structural variants alone are sufficient to alter *NAC1* transcriptional dynamics and ripening time. These experiments, along with the characterization of additional minor MD loci and potential gene×environment effects, represent the next steps towards a more complete mechanistic understanding of fruit maturity in peach.

## Supplementary Material

erag210_Supplementary_Data

## Data Availability

All data supporting the findings of this study are available in the NCBI Sequence Read Archive PRJNA1435823. Custom scripts used for data processing, analysis, and visualization are available at the GitHub repository: https://github.com/marcoc83/peachMD.

## Supplementary data

The following supplementary data are available at *JXB* online.

Fig. S1. Single-marker association analysis of maturity date in the ‘Dulcebo66’ and ‘Pulchra’ parents using 18 K SNP array markers, showing major signals on chromosome 4 in both parents.

Fig. S2. MAFFT alignment of selected ONT reads from different accessions against the ‘Lovell’ reference genome sequence across the *Md* locus, highlighting the structural variants defining the different *Md* alleles.

Fig. S3. Structural characterization and genomic reconstruction of *Md* alleles, including junction structure, breakpoints, and copy-number organization.

Fig. S4. PCR assays used to genotype *Md* alleles across selected peach accessions and segregating progenies.

Fig. S5. Discrimination of *Md* alleles using short-read sequencing data based on alignments to the *Md_WT_* reference sequence and *Md_DP_* junction.

Fig. S6. Multi-species sequence alignment of the *Md* locus showing conservation of the ancestral *Md_WT_* allele across peach and related *Prunus* species.

Fig. S7. Association between SNP Peach_AO_0443007 and maturity date based on genotype classes tested by ANOVA and Tukey’s HSD.

Table S1. Genotypic, haplotypic, and phenotypic datasets supporting the analyses performed in this study across the different populations and materials.
