# Supplementary material for: Regulatory structural variations upstream of a NAC transcription factor control fruit maturity timing in peach
Source: J Exp Bot. 2026 Apr 29;77(16):5067–82. doi: 10.1093/jxb/erag210 (PMC13529339; doi:10.1093/jxb/erag210)

Supplementary Figures S1-S7

Supplementary Figure S1. Single-marker analyses of association of maturity date (MD) in ‘Dulcebo66’ and ‘Pulchra’ parents using 2,033 and 2,826 18K-SNParray informative markers (lm × ll and nn × np according to JoinMap, respectively). Two major signals were found on chr 4 on both parents.

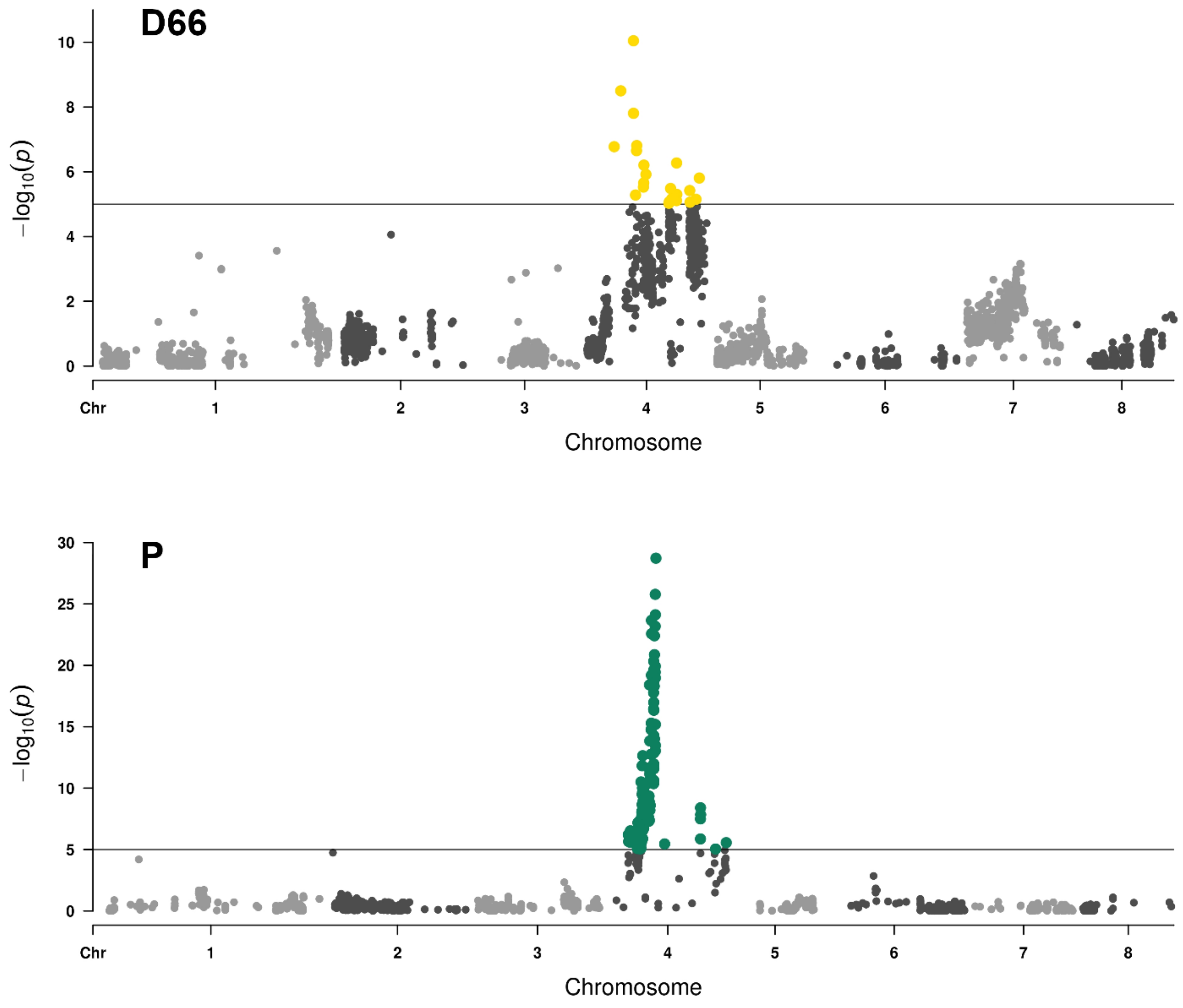

Supplementary Figure 2. MAFFT alignment of selected ONT reads of different accessions against **'**Lovell' reference genome sequence around *Md* locus. The different *Md* SVs are highlighted.

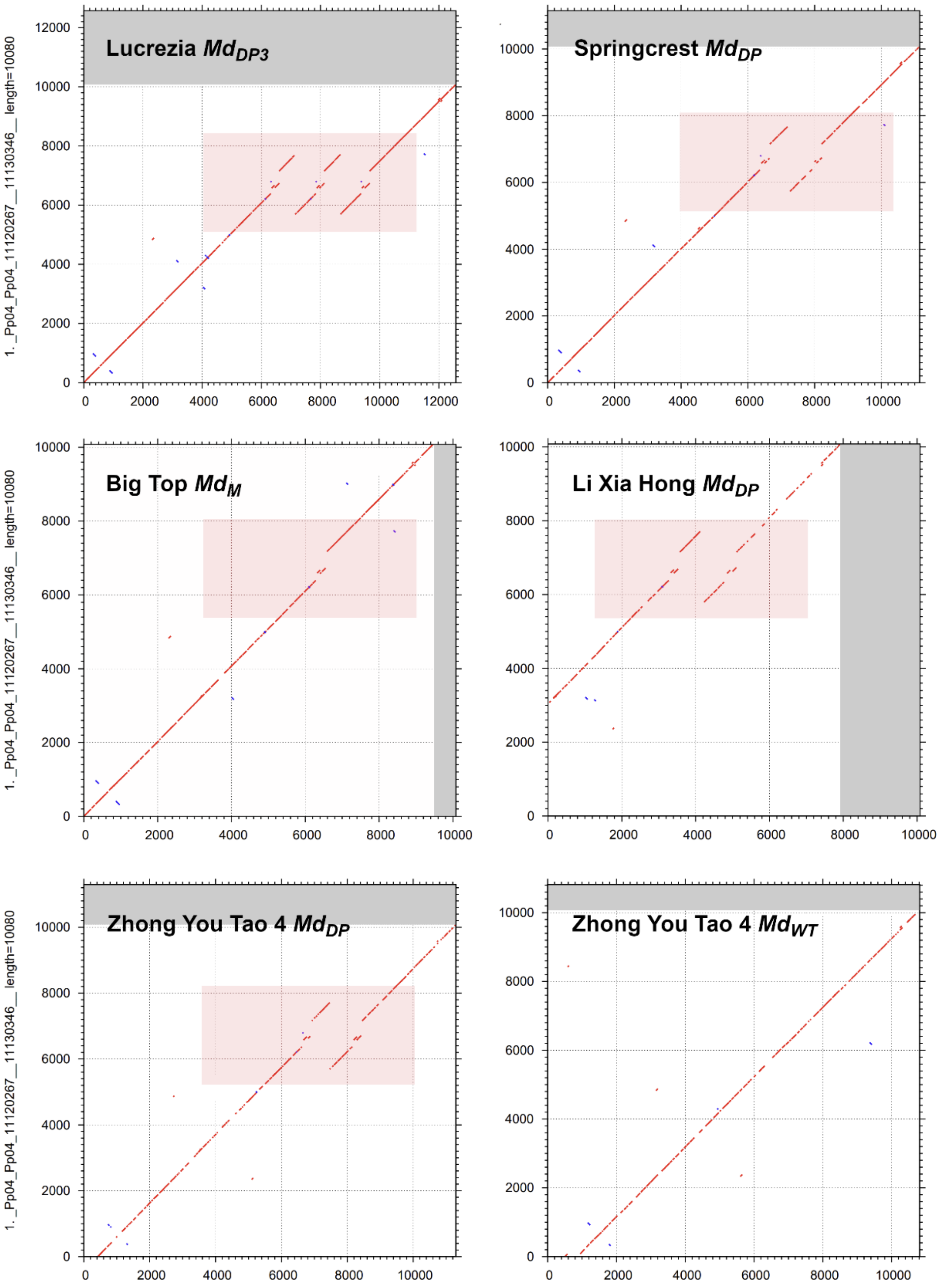

Supplementary Figure S3. Structural characterization of *Md* alleles. Genomic reconstructions of the *Md* alleles, including junction structure, breakpoints and copy-number organization.

>MdWT

TGCTAAATGAAAAGACTTGGAATCCGTCTAATTTGTATTATATTAGGAAAATGATTAATGTGTATTAATATGTGTTGCAT
-5′ UTR Prupe.4G186900
ATATATATATATATATGCAACACATATTATGACTGTCTTTGCGAAAGAAT**TTTCTGAAGCAAATCACGTCTATCACACAA**
TCCAGAATCACACTTGTATATCTCAAGCTCCTTCGGAAGTTTGGAGGCCTCCACCAATAATTCTCTATAAAATAAATGTA
GCTGGGGCACTCAAAAACGGTGATTCAATTCATAGGGGCATGTGTGGTTGTTAGAAATGAGTGTGGTGACTTTATGGGTA
GACCTGGCAATATTGTACACGATCAGTTAATACGACACGAATTCGGATCTGAGTTATCATGTTGTGTTGATATCTTGTTG
ACCCATTAGCCACACATTAAGTTAACGGGTCGTGTAAATTCGCGAATACCCCTTAACTCGTTAAACTCAGTGAAATTTTT
GTAAATTTATATCTTGTTCTTTTCTTTTTTTCTCCTTCTCTTTCTTCTCTTTCTTCTCTTCCTCCCCCATTCCCGCGAGT
CTCCCACTGTCTTTCTCTTAGTAAGTTTCTTCTCTTTTCATTATTTTTCTCAGAAACCAAACATAGCTTTCTTTTATGTT
CTTCTCTCTCGCTCTCTCACTACACAGTGGTTCTGTATGTGGTTTTTCTTTTTCTTTTTAGTTTTCATTTTTTAAATTCT
TAACAGGTGAAACAGGCAGTGTCGTGTGATTCACGAATTGTAACGGGTTGTGTTCGTATTTGACATTTTTCACTCGTTTT
CTTATCGTGTCGTGTCAACCCATCAAGAAAAGTAGGTTGACACGAACACGGCACGAACACTGCAAACACGATACGAATGC
CGGGTCTATTATGGGTGCACGCATTAAGCCAATCCGAGCCAGTTATGGGGCCAAGCAAACGGAGCTTATGGCTATTCCCG
CGAGTCTCCCACTGTCTTTCTCTTAGTAAGTTTCTTCTCTTTTCATTATTTTTCTCAGAAACCAAACATAGTTTCTTTTA
TGTTCTTCTCTCTCGCTCTCTCACTACACAGTGGTTCTGTATGTGGTTTTTCTTTTTCTTTTTAGTTTTCATTTTTTAAA
TTCTTAACAG**GTGAAACAGGCAGTGTCGTGTGATTCACGAATTGTAACGGGTTGTGTTCGTATTTGACATTTTTCACTCG**
TTTTCTTATCGTGTCGTGTCAACCCATCAAGAAAAGTAGGTTGACACGAACACGGCACGAACACTGCAAACACGATACGA
-cds Prupe.4G186900
ATGCCGGGTCTATTATGGGTGCACGCATTAAGCCAATCCGAGCCAGTTATGGGGCCAAGCAAACGGAGCTTATGGCTACA
ATTGAAGGATTGCGTTTTGCTCTTGACATGGACGTATTTGACTTGTGCCATTCTAGAGATGGACGCAAAAGAATGCATAA
-3′UTR Prupe.4G186900
CTGGTATTCTTATGACGGAGTAATGCAATGGAACATATAGTCTTTTACTTGAAGAAGCAAAATGCATATTAAGTAAATTT
AGAGTAGCTTTATGTCAATGGTCCCTAAGGATCTGCAATAAGGTGGCACACATCTTGGCTCAATTTGCAATCAGCTTTAA
TGATTTTGTTTCTTGGATTGAGGAAGCACCTACATGGTTGCTCTCATTTCTTACTCTCTTCTCGAGTTTTTGAATGAAAT
CTTGATTCTAAAATTATAAATAAAGAATATATATATTATTTATTTATGGTTTTCGTTAGATGAACAATTAATTAATGAAG
ATTAATCCTCTATTTCGTTAGATGAAATGATTTAGATATACTTTTTAATACCGAGGAAGCAGATAGGTCTTCAATCGAAG
TCAGTCATGCTAAACCTCAATTGTTTTTCAAAAGTATCACATTCATAGAAAGAGGGAGGGAAAGCTGACAAAATCTTTAT
ACAAAAGAAGCTAACTTTAATTTATATGTGGCCAGCTTGAAAGGTGCATCCGTGGCGTTGATACTTCTCATCTATTTGAT
ACCATGTATCTTTGCTCTAAAAAGTGGCCAATTTTTTGTCCTCATTTCAAAATTTGGCAAGTGATTGTACTTGACAAGAA
ATTAATATTTCCAATTTGGCTTTAAAACGACAATTAGTGAAAGCAACCCCCAACTCACAATCTGTCTCGTTCTCTGTATA
AGGCTAAGCAAGATTTGGCTACTAACACCACCTTGATTGGCTTTGTTAAGCTCTCTTCCTTGACTTATCAACACACGAAA
GATATCTTTAAGCACTAATGCAATCCTTCTCCAATGAGGGATATACTCAACAATTTTTTTGTATGTTACTATTTAGGGGA
GAACATATTTTTCATTGTAGTTTTTGTACTTATATTGACTTAATGGTGGTGACCACTTGATTAATACTAATCCGTAAACA
AAACCAGATGGAGGGCCAGCATTTTTGAATTTTTGAGTCAAGAAACAAACATTACAAAGAAGATCCTCGAGGGATATTTA
CACCATATAAATCACAATATATATACTCTATGTAAAACATTCATTGGAAAATTTCGAAACCAAATACACGTAGCTGAAGA
AACATGATCACGTTTAAACATGCTTTTAAATATACATAAAATTTAAACATCAAGGCTCAAATAAAATTATACTCAAACAA
CATGAATGTAAAATGTAATTTACAAATATTTATATTTATATATGTATGCATACTGCAAATATAGTATAATGTCTCTTGTT
TATAGATGTATGAAAGTGTGTGACATATCAACATAAATACAATTTATACATGGAAATTAGCTGTCGTGACAATGGGATTT
CGATCGAGCTGTCTCTCTCCAAATACTGAAAGCCAAATCACAGAAATGAATTATTGTCCAAATTAATCAGACCCAACTGT
CATTATAACAGGCTGTGATTTCTTAAGATTAACGTGATATGACATGCTAAGAGATCGTAGGATATTTATCAGGAAGATGA
CACATTTTGTTGATTGGATAAAAGATTTTATATGGAGAAAAAGAGAAAATACACAAGAGTTTGTGAGACTAATCCTTCTA
TTTTAAGCTCACAATTCTGGAATTGATTTGATTGATCGAAATAATATCGTCTCCTTTCAACAAAGAAATTTTTTAAAAAA
TAAAAATTAGGGCACTTATAATTTGTATCGTTGCATGGTGAGTACTTATGGATAATGTTGAGAGAATTGGGGTGTTTATT
AAGAGTGGTGTTAAACGAATCCTAAAATTAACTGAGTACACCTTACTACTAAAGTATTTATTGCATTACTAATTTACCTT
AATATAAAATAACCAAAAAGAATATATGGAATTGTAAAATAAACATAATTTTAATAAGTACATCATACTCATCATATTCA
AACCTAATCAAACGTAGAGAAATTTTCGTATTACTTGATGTCCACATCAACGCATGCGCTCTTTTAGAAAAGCCAGACAC
AGTTCAAATGTCCACAGTCCTGCTAAAGTGGTTCCCATTAAAAGAAGCCGTCTCTGTGTATATATCCATATGTTGAATAT
ATGCATCTTGTGTTGTCAATGTAACAATCAATGTTTTTAGTGTTGCTCTTTGTATTATTTTCTTTTCGCACCTTTATTTT
CTTAAGGCTTGTTTTTTTCCAAGTTTCAAAGGTTTGTACATGTATTTTGTATTTTTGGTTGAATCCGTTTTGAGTTTTCAA
TGTGTAAACTTGGGACGGTTTCAAAAAAGAAAAAAGCAAACCAAGTAGTTTCGATTTGGTTTGGTACTAACTTGTAAATC
CAGTTTTGTTAAAGGGGCACTTATTCCAAGTTGTGGAGTGATATTTACAAGTCAGATGTAGTCACTTTCGCAATCTAGAT
TAAGCTTTAGCTCATGGCATCCAATAATTTAGTGTATGTTGTGCGGCCCTGCCTTTTTATGTAATGCGTTCACCTAGTGT
TTGTTTTAACTAAGAGAAATTCAC

>MdM

TGCTAAATGAAAAGACTTGGAATCCGTCTAATTTGTATTATATTAGGAAAATGATTAATGTGTATTAATATGTGTTGCAT
-5′ UTR Prupe.4G186900

ATATATATATATATATATGCAACACATATTATGACTGTCTTTGCGAAAGAAT**TTTCTGAAGCAAATCACGTCTATCACACAA**
TCCAGAATCACACTTGTATATCTCAAGCTCCTTCGGAAGTTTGGAGGCCTCCACCAATAATTCTCTATAAAATAAATGTA
GCTGGGGCACTCAAAAACGGTGATTCAATTCATAGGGGCATGTGTGGTTGTTAGAAATGAGTGTGGTGACTTTATGGGTA
GACCTGGCAATATTGTACACGATCAGTTAATACGACACGAATTCGGATCTGAGTTATCATGTTGTGTTGATATCTTGTTG
ACCCATTAGCCACACATTAAGTTAACGGGTCGTGTAAATTCGCGAATACCCCTTAACTCGTTAAACTCAGTGAAATTTTT
GTAAATTTATATCTTGTTCTTTTCTTTTTTTCTCCTTCTCTTTCTTCTCTTTCTTCTCTTCCTCCCCCATTCCCGCGAGT
CTCCCACTGTCTTTCTCTTAGTAAGTTTCTTCTCTTTTCATTATTTTTCTCAGAAACCAAACATAGCTTTCTTTTATGTT
CTTCTCTCTCGCTCTCTCACTACACAGTGGTTCTGTATGTGGTTTTTCTTTTTCTTTTTAGTTTTCATTTTTTAAATTCT
TAACAGGTGAAACAGGCAGTGTCGTGTGATTCACGAATTGTAACGGGTTGTGTTCGTATTTGACATTTTTCACTCGTTTT
CTTATCGTGTCGTGTCAACCCATCAAGAAAAGTAGGTTGACACGAACACGGCACGAACACTGCAAACACGATACGAATGC
CGGGTCTATTATGGGTGCACGCATTAAGCCAATCCGAGCCAGTTATGGGGCCAAGCAAACGGAGCTTATGGCTATTCCCG
CGAGTCTCCCACTGTCTTTCTCTTAGTAAGTTTCTTCTCTTTTCATTATTTTTCTCAGAAACCAAACATAGTTTCTTTTA
TGTTCTTCTCTCTCGCTCTCTCACTACACAGTGGTTCTGTATGTGGTTTTTCTTTTTCTTTTTAGTTTTCATTTTTTAAA
TTCTTAACAG**GTGAAACAGGCAGTGTCGTGTGATTCACGAATTGTAACGGGTTGTGTTCGTATTTGACATTTTTCACTCG**
TTTTCTTATCGTGTCGTGTCAACCCATCAAGAAAAGTAGGTTGACACGAACACGGCACGAACACTGCAAACACGATACGA

-cds Prupe.4G186900

ATGCCGGGTCTATTATGGGTGCACGCATTAAGCCAATCCGAGCCAGTTATGGGGCCAAGCAAACGGAGCTTATGGCTACA
ATTGAAGGATTGCGTTTTGCTCTTGACATGGACGTATTTGACTTGTGCCATTCTAGAGATGGACGCAAAAGAATGCATAA

-3’UTR Prupe.4G186900

CTGGTATTCTTATGACGGAGTAATGCAATGGAACATATAGTCTTTTACTTGAAGAAGCAAAATGCATATTAAGTAAATTT
AGAGTAGCTTTATGTCAATGGTCCCTAAGGATCTGCAATAAGGTGGCACACATCTTGGCTCAATTTGCAATCAGCTTTAA
TGATTTTGTTTCTTGGATTGAGGAAGCACCTACATGGTTGCTCTCATTTCTTACTCTCTTCTCGAGTTTTTGAATGAAAT
CTTGATTCTAAAATTATAAATAAAGAATATATATATTATTTATTTATGGTTTTCGTTAGATGAACAATTAATTAATGAAG
ATTAATCCTCTATTTCGTTAGATGAAATGATTTAGATATACTTTTTAATACCGAGGAAGCAGATAGGTCTTCAATCGAAG

Pos. 11,126,654 **94-bp tandem dup/ins**

TCAGTCATGCTAAACCTCAATTGTTTTTCAAAAGTATCACATTCATAG***TAACATACAAAAAAATTGTTGAGTATATTTCC***
AAGAAATTAATATTTCCAATTTGGCTTTAAAACGACAATTAGTGAAAGCAACCCCCAACTCACAATCTGTCTCGTTCTCT
GTATAAGGCTAAG***CAAGAAATTAATATTTCCAATTTGGCTTTAAAACGACAATTAGTGAAAGCAACCCCCAACTCACAAT***
CTGTCTCGTTCTCTGTATAAGGCTAAGCAAGATTTGGCTACTAACACCACCTTGATTGGCTTTGTTAAGCTCTCTTCCTT

-400 bp deletion in M allele

GACTTATCAACACACGAAAGATA**~~TCTTTAAGCACTAATGCAATCCTTCTCCAATGAGGGATATACTCAACAATTTTTTTG~~**
~~TATGTTACTATTTAGGGGAGAACATATTTTTCATTGTAGTTTTTGTACTTATATTGACTTAATGGTGGTGACCACTTGAT~~
~~TAATACTAATCCGTAAACAAAACCAGATGGAGGGCCAGCATTTTTGAATTTTTGAGTCAAGAAACAAACATTACAAAGAA~~
~~GATCCTCGAGGGATATTTACACCATATAAATCACAATATATATACTCTATGTAAAACATTCATTGGAAAATTTCGAAACC~~
~~AAATACACGTAGCTGAAGAAACATGATCACGTTTAAACATGCTTTTAAATATACATAAAATTTAAACATCAAGGCTCAAA~~
~~TAAAATTATACTCAAACAACATGAATGTAAAATGTA~~ATTTACAAATATTTATATTTATATATGTATGCATACTGCAAATA
TAGTATAATGTCTCTTGTTTATAGATGTATGAAAGTGTGTGACATATCAACATAAATACAATTTATACATGGAAATTAGC
TGTCGTGACAATGGGATTTCGATCGAGCTGTCTCTCTCCAAATACTGAAAGCCAAATCACAGAAATGAATTATTGTCCAA
ATTAATCAGACCCAACTGTCATTATAACAGGCTGTGATTTCTTAAGATTAACGTGATATGACATGCTAAGAGATCGTAGG
ATATTTATCAGGAAGATGACACATTTTGTTGATTGGATAAAAGATTTTATATGGAGAAAAAGAGAAAATACACAAGAGTT
TGTGAGACTAATCCTTCTATTTTAAGCTCACAATTCTGGAATTGATTTGATTGATCGAAATAATATCGTCTCCTTTCAAC
AAAGAAATTTTTTAAAAAATAAAAATTAGGGCACTTATAATTTGTATCGTTGCATGGTGAGTACTTATGGATAATGTTGA
GAGAATTGGGGTGTTTATTAAGAGTGGTGTTAAACGAATCCTAAAATTAACTGAGTACACCTTACTACTAAAGTATTTAT
TGCATTACTAATTTACCTTAATATAAAATAACCAAAAAGAATATATGGAATTGTAAAATAAACATAATTTTAATAAGTAC
ATCATACTCATCATATTCAAACCTAATCAAACGTAGAGAAATTTTCGTATTACTTGATGTCCACATCAACGCATGCGCTC
TTTTAGAAAAGCCAGACACAGTTCAAATGTCCACAGTCCTGCTAAAGTGGTTCCCATTAAAAGAAGCCGTCTCTGTGTAT
ATATCCATATGTTGAATATATGCATCTTGTGTTGTCAATGTAACAATCAATGTTTTTAGTGTTGCTCTTTGTATTATTTT
CTTTTCGCACCTTTATTTTCTTAAGGCTTGTTTTTTCCAAGTTTCAAAGGTTTGTACATGTATTTTGTATTTTTGGTTGA
ATCCGTTTTGAGTTTTCAATGTGTAAACTTGGGACGGTTTCAAAAAAGAAAAAAGCAAACCAAGTAGTTTCGATTTGGTT
TGGTACTAACTTGTAAATCCAGTTTTGTTAAAGGGGCACTTATTCCAAGTTGTGGAGTGATATTTACAAGTCAGATGTAG
TCACTTTCGCAATCTAGATTAAGCTTTAGCTCATGGCATCCAATAATTTAGTGTATGTTGTGCGGCCCTGCCTTTTTATG
TAATGCGTTCACCTAGTGTTTGTTTTAACTAAGAGAAATTCAC

>MdDP

TGCTAAATGAAAAGACTTGGAATCCGTCTAATTTGTATTATATTAGGAAAATGATTAATGTGTATTAATATGTGTTGCAT
ATATATATATATATATATGCAACACATATTATGACTGTCTTTGCGAAAGAAT**TTTCTGAAGCAAATCACGTCTATCACACAA**
TCCAGAATCACACTTGTATATCTCAAGCTCCTTCGGAAGTTTGGAGGCCTCCACCAATAATTCTCTATAAAATAAATGTA
GCTGGGGCACTCAAAAACGGTGATTCAATTCATAGGGGCATGTGTGGTTGTTAGAAATGAGTGTGGTGACTTTATGGGTA
GACCTGGCAATATTGTACACGATCAGTTAATACGACACGAATTCGGATCTGAGTTATCATGTTGTGTTGATATCTTGTTG
ACCCATTAGCCACACATTAAGTTAACGGGTCGTGTAAATTCGCGAATACCCCTTAACTCGTTAAACTCAGTGAAATTTTT
GTAAATTTATATCTTGTTCTTTTCTTTTTTTCTCCTTCTCTTTCTTCTCTTTCTTCTCTTCCTCCCCCATTCCCGCGAGT
CTCCCACTGTCTTTCTCTTAGTAAGTTTCTTCTCTTTTCATTATTTTTCTCAGAAACCAAACATAGCTTTCTTTTATGTT

CTTCTCTCTCGCTCTCTCACTACACAGTGGTTCTGTATGTGGTTTTTCTTTTTCTTTTTAGTTTTCATTTTTTAAATTCT
TAACAGGTGAAACAGGCAGTGTCGTGTGATTCACGAATTGTAACGGGTTGTGTTCGTATTTGACATTTTTCACTCGTTTT
CTTATCGTGTCGTGTCAACCCATCAAGAAAAGTAGGTTGACACGAACACGGCACGAACACTGCAAACACGATACGAATGC
CGGGTCTATTATGGGTGCACGCATTAAGCCAATCCGAGCCAGTTATGGGGCCAAGCAAACGGAGCTTATGGCTATTCCCG
CGAGTCTCCCACTGTCTTTCTCTTAGTAAGTTTCTTCTCTTTTCATTATTTTTCTCAGAAACCAAACATAGTTTCTTTTA
TGTTCTTCTCTCTCGCTCTCTCACTACACAGTGGTTCTGTATGTGGTTTTTCTTTTTCTTTTTAGTTTTCATTTTTTAAA
TTCTTAACAG**GTGAAACAGGCAGTGTCGTGTGATTCACGAATTGTAACGGGTTGTGTTCGTATTTGACATTTTTCACTCG**
TTTTCTTATCGTGTCGTGTCAACCCATCAAGAAAAGTAGGTTGACACGAACACGGCACGAACACTGCAAACACGATACGA
ATGCCGGGTCTATTATGGGTGCACGCATTAAGCCAATCCGAGCCAGTTATGGGGCCAAGCAAACGGAGCTTATGGCTACA
ATTGAAGGATTGCGTTTTGCTCTTGACATGGACGTATTTGACTTGTGCCATTCTAGAGATGGACGCAAAAGAATGCATAA
CTGGTATTCTTATGACGGAGTAATGCAATGGAACATATAGTCTTTTACTTGAAGAAGCAAAATGCATATTAAGTAAATTT
AGAGTAGCTTTATGTCAATGGTCCCTAAGGATCTGCAATAAGGTGGCACACATCTTGGCTCAATTTGCAATCAGCTTTAA
TGATTTTGTTTCTTGGATTGAGGAAGCACCTACATGGTTGCTCTCATTTCTTACTCTCTTCTCGAGTTTTTGAATGAAAT
CTTGATTCTAAAATTATAAATAAAGAATATATATATTATTTATTTATGGTTTTCGTTAGATGAACAATTAATTAATGAAG
ATTAATCCTCTATTTCGTTAGATGAAATGATTTAGATATACTTTTTAATACCGAGGAAGCAGATAGGTCTTCAATCGAAG
TCAGTCATGCTAAACCTCAATTGTTTTTCAAAAGTATCACATTCATAG***TAACATACAAAAAAATTGTTGAGTATATTTCC***
AAGAAATTAATATTTCCAATTTGGCTTTAAAACGACAATTAGTGAAAGCAACCCCCAACTCACAATCTGTCTCGTTCTCT
GTATAAGGCTAAGCAAGAAATTAATATTTCCAATTTGGCTTTAAAACGACAATTAGTGAAAGCAACCCCCAACTCACAAT
CTGTCTCGTTCTCTGTATAAGGCTAAGCAAGATTTGGCTACTAACACCACCTTGATTGGCTTTGTTAAGCTCTCTTCCTT
GACTTATCAACACACGAAAGATAATTTACAAATATTTATATTTATATATGTATGCATACTGCAAATATAGTATAATGTCT
CTTGTTTATAGATGTATGAAAGTGTGTGACATATCAACATAAATACAATTTATACATGGAAATTAGCTGTCGTGACAATG
GGATTTCGATCGAGCTGTCTCTCTCCAAATACTGAAAGCCAAATCACAGAAATGAATTATTGTCCAAATTAATCAGACCC
AACTGTCATTATAACAGGCTGTGATTTCTTAAGATTAACGTGATATGACATGCTAAGAGATCGTAGGATATTTATCAGGA
AGATGACACATTTTGTTGATTGGATAAAAGATTTTATATGGAGAAAAAGAGAAAATACACAAGAGTTTGTGAGACTAATC
CTTCTATTTTAAGCTCACAATTCTGGAATTGATTTGATTGATCGAAATAATATCGTCTCCTTTCAACAAAGAAATTTTTT
AAAAAATAAAAATTAGGGCACTTATAATTTGTATCGTTGCATGGTGAGTACTTATGGATAATGTTGAGAGAATTGGGGTG

DUPLICATION_START

TTTATTAAGAGTGGTGTT***TTTCACTCGTTTTCTTATCGTGTCGTGTCAACCCATCAAGAAAAGTAGGTTGACACGAACAC***
GGCACGAACACTGCAAACACGATACGAATGCCGGGTCTATTATGGGTGCACGCATTAAGCCAATCCGAGCCAGTTATGGG
GCCAAGCAAACGGAGCTTATGGCTACAATTGAAGGATTGCGTTTTGCTCTTGACATGGACGTATTTGACTTGTGCCATTC
TAGAGATGGACGCAAAAGAATGCATAACTGGTATTCTTATGACGGAGTAATGCAATGGAACATATAGTCTTTTACTTGAA
GAAGCAAAATGCATATTAAGTAAATTTAGAGTAGCTTTATGTCAATGGTCCCTAAGGATCTGCAATAAGGTGGCACACAT
CTTGGCTCAATTTGCAATCAGCTTTAATGATTTTGTTTCTTGGATTGAGGAAGCACCTACATGGTTGCTCTCATTTCTTA
CT*CTCTTCTCGAGTTTTTGAATGAAATCTTGATTCTAAAATTATAAATAAAGAATATATATATTATTTATTTATGGTTTT*
CGTTAGATGAACAATTAATTAATGAAGATTAATCCTCTATTTCGTTAGATGAAATGATTTAGATATACTTTTTAATACCG
*AGGAAGCAGATAGGTCTTCAATCGAAGTCAGTCATGCTAAACCTCAATTGTTTTTCAAAAGTATCACATTCATAG****TAACA***
TACAAAAAAATTGTTGAGTATATTTCCAAGAAATTAATATTTCCAATTTGGCTTTAAAACGACAATTAGTGAAAGCAACC
CCCAACTCACAATCTGTCTCGTTCTCTGTATAAGGCTAAGCAAGAAATTAATATTTCCAATTTGGCTTTAAAACGACAAT
TAGTGAAAGCAACCCCCAACTCACAATCTGTCTCGTTCTCTGTATAAGGCTAAG*CAAGATTTGGCTACTAACACCACCTT*
GATTGGCTTTGTTAAGCTCTCTTCCTTGACTTATCAACACACGAAAGATAATTTACAAATATTTATATTTATATATGTAT
GCATACTGCAAATATAGTATAATGTCTCTTGTTTATAGATGTATGAAAGTGTGTGACATATCAACATAAATACAATTTAT
ACATGGAAATTAGCTGTCGTGACAATGGGATTTCGATCGAGCTGTCTCTCTCCAAATACTGAAAGCCAAATCACAGAAAT
GAATTATTGTCCAAATTAATCAGACCCAACTGTCATTATAACAGGCTGTGATTTCTTAAGATTAACGTGATATGACATGC
TAAGAGATCGTAGGATATTTATCAGGAAGATGACACATTTTGTTGATTGGATAAAAGATTTTATATGGAGAAAAAGAGAA
AATACACAAGAGTTTGTGAGACTAATCCTTCTATTTTAAGCTCACAATTCTGGAATTGATTTGATTGATCGAAATAATAT
CGTCTCCTTTCAACAAAGAAATTTTTTAAAAAATAAAAATTAGGGCACTTATAATTTGTATCGTTGCATGGTGAGTACTT

DUPLICATION_END

*ATGGATAATGTTGAGAGAATTGGGGTG****TTTATTAAGAGTGGTGTT***AAACGAATCCTAAAATTAACTGAGTACACCTTACT
ACTAAAGTATTTATTGCATTACTAATTTACCTTAATATAAAATAACCAAAAAGAATATATGGAATTGTAAAATAAACATA
ATTTTAATAAGTACATCATACTCATCATATTCAAACCTAATCAAACGTAGAGAAATTTTCGTATTACTTGATGTCCACAT
CAACGCATGCGCTCTTTTAGAAAAGCCAGACACAGTTCAAATGTCCACAGTCCTGCTAAAGTGGTTCCCATTAAAAGAAG
CCGTCTCTGTGTATATATCCATATGTTGAATATATGCATCTTGTGTTGTCAATGTAACAATCAATGTTTTTAGTGTTGCT
CTTTGTATTATTTTCTTTTCGCACCTTTATTTTCTTAAGGCTTGTTTTTTCCAAGTTTCAAAGGTTTGTACATGTATTTT
GTATTTTTGGTTGAATCCGTTTTGAGTTTTCAATGTGTAAACTTGGGACGGTTTCAAAAAAGAAAAAAGCAAACCAAGTA
GTTTCGATTTGGTTTGGTACTAACTTGTAAATCCAGTTTTGTTAAAGGGGCACTTATTCCAAGTTGTGGAGTGATATTTA
CAAGTCAGATGTAGTCACTTTCGCAATCTAGATTAAGCTTTAGCTCATGGCATCCAATAATTTAGTGTATGTTGTGCGGC
CCTGCCTTTTTATGTAATGCGTTCACCTAGTGTTTGTTTTAACTAAGAGAAATTCAC

>MdDP3

TGCTAAATGAAAAGACTTGGAATCCGTCTAATTTGTATTATATTAGGAAAATGATTAATGTGTATTAATATGTGTTGCAT
ATATATATATATATATGCAACACATATTATGACTGTCTTTGCGAAAGAAT**TTTCTGAAGCAAATCACGTCTATCACACAA**
TCCAGAATCACACTTGTATATCTCAAGCTCCTTCGGAAGTTTGGAGGCCTCCACCAATAATTCTCTATAAAATAAATGTA

GCTGGGGCACTCAAAAACGGTGATTCAATTCATAGGGGCATGTGTGGTTGTTAGAAATGAGTGTGGTGACTTTATGGGTA
GACCTGGCAATATTGTACACGATCAGTTAATACGACACGAATTCGGATCTGAGTTATCATGTTGTGTTGATATCTTGTTG
ACCCATTAGCCACACATTAAGTTAACGGGTCGTGTAAATTCGCGAATACCCCTTAACTCGTTAAACTCAGTGAAATTTTT
GTAAATTTATATCTTGTTCTTTTCTTTTTTTCTCCTTCTCTTTCTTCTCTTTCTTCTCTTCCTCCCCCATTCCCGCGAGT
CTCCCACTGTCTTTCTCTTAGTAAGTTTCTTCTCTTTTCATTATTTTTCTCAGAAACCAAACATAGCTTTCTTTTATGTT
CTTCTCTCTCGCTCTCTCACTACACAGTGGTTCTGTATGTGGTTTTTCTTTTTCTTTTTAGTTTTCATTTTTTAAATTCT
TAACAGGTGAAACAGGCAGTGTCGTGTGATTCACGAATTGTAACGGGTTGTGTTCGTATTTGACATTTTTCACTCGTTTT
CTTATCGTGTCGTGTCAACCCATCAAGAAAAGTAGGTTGACACGAACACGGCACGAACACTGCAAACACGATACGAATGC
CGGGTCTATTATGGGTGCACGCATTAAGCCAATCCGAGCCAGTTATGGGGCCAAGCAAACGGAGCTTATGGCTATTCCCG
CGAGTCTCCCACTGTCTTTCTCTTAGTAAGTTTCTTCTCTTTTCATTATTTTTCTCAGAAACCAAACATAGTTTCTTTTA
TGTTCTTCTCTCTCGCTCTCTCACTACACAGTGGTTCTGTATGTGGTTTTTCTTTTTCTTTTTAGTTTTCATTTTTTAAA
TTCTTAACAGGTGAAACAGGCAGTGTCGTGTGATTCACGAATTGTAACGGGTTGTGTTCGTATTTGACATTTTTCACTCG
TTTTCTTATCGTGTCGTGTCAACCCATCAAGAAAAGTAGGTTGACACGAACACGGCACGAACACTGCAAACACGATACGA
ATGCCGGGTCTATTATGGGTGCACGCATTAAGCCAATCCGAGCCAGTTATGGGGCCAAGCAAACGGAGCTTATGGCTACA
ATTGAAGGATTGCGTTTTGCTCTTGACATGGACGTATTTGACTTGTGCCATTCTAGAGATGGACGCAAAAGAATGCATAA
CTGGTATTCTTATGACGGAGTAATGCAATGGAACATATAGTCTTTTACTTGAAGAAGCAAAATGCATATTAAGTAAATTT
AGAGTAGCTTTATGTCAATGGTCCCTAAGGATCTGCAATAAGGTGGCACACATCTTGGCTCAATTTGCAATCAGCTTTAA
TGATTTTGTTTCTTGGATTGAGGAAGCACCTACATGGTTGCTCTCATTTCTTACTCTCTTCTCGAGTTTTTGAATGAAAT
CTTGATTCTAAAATTATAAATAAAGAATATATATATTATTTATTTATGGTTTTCGTTAGATGAACAATTAATTAATGAAG
ATTAATCCTCTATTTCGTTAGATGAAATGATTTAGATATACTTTTTAATACCGAGGAAGCAGATAGGTCTTCAATCGAAG
TCAGTCATGCTAAACCTCAATTGTTTTTCAAAAGTATCACATTCATAGTAACATACAAAAAAATTGTTGAGTATATTTCC
AAGAAATTAATATTTCCAATTTGGCTTTAAAACGACAATTAGTGAAAGCAACCCCCAACTCACAATCTGTCTCGTTCTCT
GTATAAGGCTAAGCAAGAAATTAATATTTCCAATTTGGCTTTAAAACGACAATTAGTGAAAGCAACCCCCAACTCACAAT
CTGTCTCGTTCTCTGTATAAGGCTAAGCAAGATTTGGCTACTAACACCACCTTGATTGGCTTTGTTAAGCTCTCTTCCTT
GACTTATCAACACACGAAAGATAATTTACAAATATTTATATTTATATATGTATGCATACTGCAAATATAGTATAATGTCT
CTTGTTTATAGATGTATGAAAGTGTGTGACATATCAACATAAATACAATTTATACATGGAAATTAGCTGTCGTGACAATG
GGATTTCGATCGAGCTGTCTCTCTCCAAATACTGAAAGCCAAATCACAGAAATGAATTATTGTCCAAATTAATCAGACCC
AACTGTCATTATAACAGGCTGTGATTTCTTAAGATTAACGTGATATGACATGCTAAGAGATCGTAGGATATTTATCAGGA
AGATGACACATTTTGTTGATTGGATAAAAGATTTTATATGGAGAAAAAGAGAAAATACACAAGAGTTTGTGAGACTAATC
CTTCTATTTTAAGCTCACAATTCTGGAATTGATTTGATTGATCGAAATAATATCGTCTCCTTTCAACAAAGAAATTTTTT
AAAAAATAAAAATTAGGGCACTTATAATTTGTATCGTTGCATGGTGAGTACTTATGGATAATGTTGAGAGAATTGGGGTG

DUPLICATION_START   -5′UTR Prupe.4G186900

TTTATTAAGAGTGGTGTTTTTCACTCGTTTTCTTATCGTGTCGTGTCAACCCATCAAGAAAAGTAGGTTGACACGAACAC
GGCACGAACACTGCAAACACGATACGAATGCCGGGTCTATTATGGGTGCACGCATTAAGCCAATCCGAGCCAGTTATGGG
GCCAAGCAAACGGAGCTTATGGCTACAATTGAAGGATTGCGTTTTGCTCTTGACATGGACGTATTTGACTTGTGCCATTC
TAGAGATGGACGCAAAAGAATGCATAACTGGTATTCTTATGACGGAGTAATGCAATGGAACATATAGTCTTTTACTTGAA
GAAGCAAAATGCATATTAAGTAAATTTAGAGTAGCTTTATGTCAATGGTCCCTAAGGATCTGCAATAAGGTGGCACACAT
CTTGGCTCAATTTGCAATCAGCTTTAATGATTTTGTTTCTTGGATTGAGGAAGCACCTACATGGTTGCTCTCATTTCTTA
CTCTCTTCTCGAGTTTTTGAATGAAATCTTGATTCTAAAATTATAAATAAAGAATATATATATTATTTATTTATGGTTTT
CGTTAGATGAACAATTAATTAATGAAGATTAATCCTCTATTTCGTTAGATGAAATGATTTAGATATACTTTTTAATACCG
AGGAAGCAGATAGGTCTTCAATCGAAGTCAGTCATGCTAAACCTCAATTGTTTTTCAAAAGTATCACATTCATAGTAACA
TACAAAAAAATTGTTGAGTATATTTCCAAGAAATTAATATTTCCAATTTGGCTTTAAAACGACAATTAGTGAAAGCAACC
CCCAACTCACAATCTGTCTCGTTCTCTGTATAAGGCTAAGCAAGAAATTAATATTTCCAATTTGGCTTTAAAACGACAAT
TAGTGAAAGCAACCCCCAACTCACAATCTGTCTCGTTCTCTGTATAAGGCTAAGCAAGATTTGGCTACTAACACCACCTT
GATTGGCTTTGTTAAGCTCTCTTCCTTGACTTATCAACACACGAAAGATAATTTACAAATATTTATATTTATATATGTAT
GCATACTGCAAATATAGTATAATGTCTCTTGTTTATAGATGTATGAAAGTGTGTGACATATCAACATAAATACAATTTAT
ACATGGAAATTAGCTGTCGTGACAATGGGATTTCGATCGAGCTGTCTCTCTCCAAATACTGAAAGCCAAATCACAGAAAT
GAATTATTGTCCAAATTAATCAGACCCAACTGTCATTATAACAGGCTGTGATTTCTTAAGATTAACGTGATATGACATGC
TAAGAGATCGTAGGATATTTATCAGGAAGATGACACATTTTGTTGATTGGATAAAAGATTTTATATGGAGAAAAAGAGAA
AATACACAAGAGTTTGTGAGACTAATCCTTCTATTTTAAGCTCACAATTCTGGAATTGATTTGATTGATCGAAATAATAT
CGTCTCCTTTCAACAAAGAAATTTTTTAAAAAATAAAAATTAGGGCACTTATAATTTGTATCGTTGCATGGTGAGTACTT

END2xDUP_START

ATGGATAATGTTGAGAGAATTGGGGTGTTTATTAAGAGTGGTGTTTTTCACTCGTTTTCTTATCGTGTCGTGTCAACCCA
TCAAGAAAAGTAGGTTGACACGAACACGGCACGAACACTGCAAACACGATACGAATGCCGGGTCTATTATGGGTGCACGC
ATTAAGCCAATCCGAGCCAGTTATGGGGCCAAGCAAACGGAGCTTATGGCTACAATTGAAGGATTGCGTTTTGCTCTTGA
CATGGACGTATTTGACTTGTGCCATTCTAGAGATGGACGCAAAAGAATGCATAACTGGTATTCTTATGACGGAGTAATGC
AATGGAACATATAGTCTTTTACTTGAAGAAGCAAAATGCATATTAAGTAAATTTAGAGTAGCTTTATGTCAATGGTCCCT
AAGGATCTGCAATAAGGTGGCACACATCTTGGCTCAATTTGCAATCAGCTTTAATGATTTTGTTTCTTGGATTGAGGAAG
CACCTACATGGTTGCTCTCATTTCTTACTCTCTTCTCGAGTTTTTGAATGAAATCTTGATTCTAAAATTATAAATAAAGA
ATATATATATTATTTATTTATGGTTTTCGTTAGATGAACAATTAATTAATGAAGATTAATCCTCTATTTCGTTAGATGAA
ATGATTTAGATATACTTTTTAATACCGAGGAAGCAGATAGGTCTTCAATCGAAGTCAGTCATGCTAAACCTCAATTGTTT
TTCAAAAGTATCACATTCATAGTAACATACAAAAAAATTGTTGAGTATATTTCCAAGAAATTAATATTTCCAATTTGGCT

TTAAAACGACAATTAGTGAAAGCAACCCCCAACTCACAATCTGTCTCGTTCTCTGTATAAGGCTAAGCAAGAAATTAATA
TTTCCAATTTGGCTTTAAAACGACAATTAGTGAAAGCAACCCCCAACTCACAATCTGTCTCGTTCTCTGTATAAGGCTAA
G_CAAGATTTGGCTACTAACACCACCTTGATTGGCTTTGTTAAGCTCTCTTCCTTGACTTATCAACACACGAAAGATAATT_
TACAAATATTTATATTTATATATGTATGCATACTGCAAATATAGTATAATGTCTCTTGTTTATAGATGTATGAAAGTGTG
TGACATATCAACATAAATACAATTTATACATGGAAATTAGCTGTCGTGACAATGGGATTTCGATCGAGCTGTCTCTCTCC
AAATACTGAAAGCCAAATCACAGAAATGAATTATTGTCCAAATTAATCAGACCCAACTGTCATTATAACAGGCTGTGATT
TCTTAAGATTAACGTGATATGACATGCTAAGAGATCGTAGGATATTTATCAGGAAGATGACACATTTTGTTGATTGGATA
AAAGATTTTATATGGAGAAAAAGAGAAAATACACAAGAGTTTGTGAGACTAATCCTTCTATTTTAAGCTCACAATTCTGG
AATTGATTTGATTGATCGAAATAATATCGTCTCCTTTCAACAAAGAAATTTTTTAAAAAATAAAAATTAGGGCACTTATA

2xDUPLICATION_END

ATTTGTATCGTTGCATGGTGAGTACTTATGGATAATGTTGAGAGAATTGGGGTG***TTTATTAAGAGTGGTGTT***AAACGAAT
CCTAAAATTAACTGAGTACACCTTACTACTAAAGTATTTATTGCATTACTAATTTACCTTAATATAAAATAACCAAAAAG
AATATATGGAATTGTAAAATAAACATAATTTTAATAAGTACATCATACTCATCATATTCAAACCTAATCAAACGTAGAGA
AATTTTCGTATTACTTGATGTCCACATCAACGCATGCGCTCTTTTAGAAAAGCCAGACACAGTTCAAATGTCCACAGTCC
TGCTAAAGTGGTTCCCATTAAAAGAAGCCGTCTCTGTGTATATATCCATATGTTGAATATATGCATCTTGTGTTGTCAAT
GTAACAATCAATGTTTTTAGTGTTGCTCTTTGTATTATTTTCTTTTCGCACCTTTATTTTCTTAAGGCTTGTTTTTTCCA
AGTTTCAAAGGTTTGTACATGTATTTTGTATTTTTGGTTGAATCCGTTTTGAGTTTTCAATGTGTAAACTTGGGACGGTT
TCAAAAAAGAAAAAAGCAAACCAAGTAGTTTCGATTTGGTTTGGTACTAACTTGTAAATCCAGTTTTGTTAAAGGGGCAC
TTATTCCAAGTTGTGGAGTGATATTTACAAGTCAGATGTAGTCACTTTCGCAATCTAGATTAAGCTTTAGCTCATGGCAT
CCAATAATTTAGTGTATGTTGTGCGGCCCTGCCTTTTTATGTAATGCGTTCACCTAGTGTTTGTTTTAACTAAGAGAAAT
TCAC

Supplementary Figure 4. PCR assays to genotype *Md* alleles. In panel A: WT marker in accessions: Springcrest (Sp), Big Top (Bt), Lucrezia (L), Pulchra (P), Dulcebo66 (D), Elegant Lady (El), Maycrest (M) and Mayflower (Mf). Amplification is visible only in Sp, D and El. In panel B: DP marker using the same species panel as in (A). In this case, bands are observed in Sp, P, L, M and Mf. In C: M/WT marker on Sp, Bt, L, P, and D. In D: screening of Bt x Ak (1 – 10) and E x E (11 – 20) progenies with DP marker.

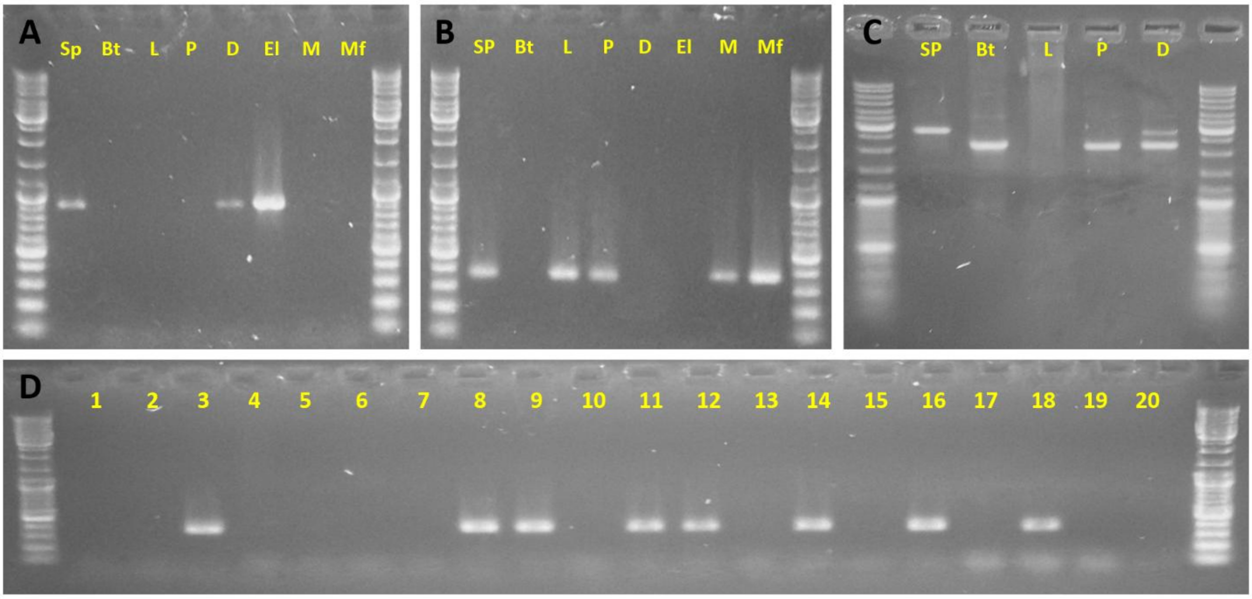

Supplementary Figure 5. *Md* allele discrimination using short reads. Alignments to the reference genome Md_{WT} sequence and Md_{DP} junction allow discrimination of Md_{WT}, Md_{M}, and Md_{DP}, whereas Md_{DP3} cannot be resolved from Md_{DP} using short reads. *Sr* allele was identified by whole-genome realignment. Shown are representative patterns for six cultivars.

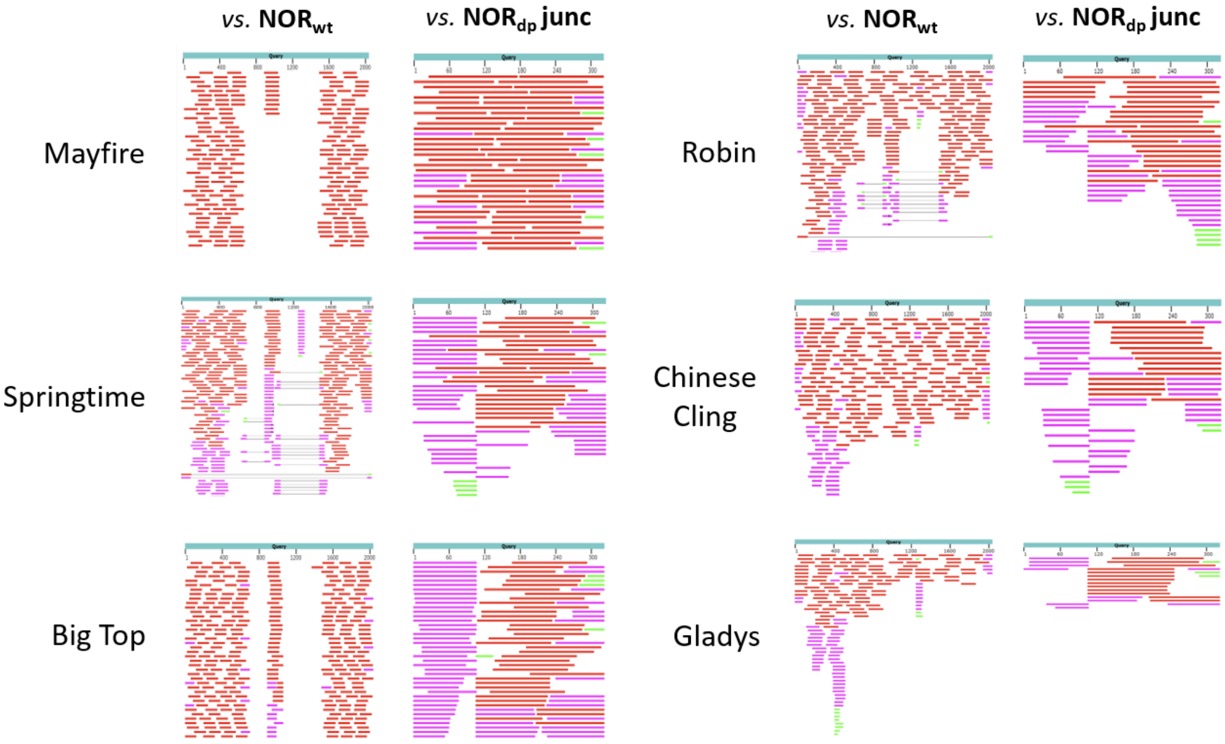

Supplementary Figure S6. Multi-species alignment of the *Md* locus. Comparative sequence alignment using Md_{WT} allele across peach and wild *Prunus* relatives shows strong conservation and confirms Md_{WT} as the ancestral allele. Structural variants found in cultivated peach are absent in wild species.

```
            1                                                                                                                                             130
Pd04:11477     CACTCGT TTTCTTATCA TGTCGTGTCA ACCCATCAAG AAAAGTAGGT TGACACGAAC ACGACACGAA CACTGCAAAC ATGATACGAA TGCCAGGTCT ATTTATGGGT GCATGCATTA AGCCAATCCG
Pp04:11125  TTTCACTCGT TTTCTTATCG TGTCGTGTCA ACCCATCAAG AAAAGTAGGT TGACACGAAC ACGGCACGAA CACTGCAAAC ACGATACGAA TGCCGGGTCT ATT-ATGGGT GCACGCATTA AGCCAATCCG
Pka04:1145   TTCACTCGT TTTCTTATCG TGTCGTGTCA ACCCATCAAG AAAAGTAGGT TGACACGAAC ACGACACGAA CACTGCAAAC ACGATATGAA TGCCGGGTCT ATT-ATGGGT GCACGCATTA AGCCAATCCG
Pda04:1175    CCACTCGT TTTCTTATCG TGTCGTGTCA ACCCATCAAG AAAAGTAGGT TGACACGAAC ACGACACGAA CACTGCAAAC ACGATACGAA TGTCGGGTCT ATTTATGGGT GCACGCATTA AGCCAATCCG
Pmi04:1191  TTTCACTCAT TTTCTTATCG TGTCGTGTCA ACCCATCAAG AAAAGTAGGT TGACACGAAC ACGACACGAA CACTGCAAAC ACGATACGAA TGCCGGGTCT ATTTATGGGT GCACGCATTA AGCCAATCCG
Pam_chr4:1                    TCG TGTCGTGTCA ACCTGTTACA AAAAGCAGGT TGACACGAAC ACGGCACAAA CACTGCAAAC ACGACACGAA TGCCAGATCT ATTTATCGGT GCACGCGTTA AGCCAATCCA
            131                                                                                                                                           260
Pd04:11477  AGCCAGTTAT GGGGCCAAGC AAACGGAGCT TATGGCTACA ATTGAAGGAT TGCGTTTTGC TCTTGACATG GACGTATTTG ACTTGTGCTA TTCTAGAGAT GGACGCAAAA GAATGCATAA CTGGTATTCT
Pp04:11125  AGCCAGTTAT GGGGCCAAGC AAACGGAGCT TATGGCTACA ATTGAAGGAT TGCGTTTTGC TCTTGACATG GACGTATTTG ACTTGTGCCA TTCTAGAGAT GGACGCAAAA GAATGCATAA CTGGTATTCT
Pka04:1145  AGCCAGTTAT GGGGCCAAGC AAACGGAGCT TATGGCTACA ATTGAAGGAT TGCGTTTTGC TCTTGACATG GACGTATTTG ACTTGTGCCA TTCTAGAGAT GGACGCAAAA GAATGCATAA CTGGTATTCT
Pda04:1175  AGCCAGTTAT GGGGCCAAGC AAACGGAGCT TATGGCTACA ATTGAAGGAT TGCGTTTTGC TCTTGACATG GACGTATTTG ACTTGTGCCA TTCTAGAGAT GGACGCAAAA GAATGCATAA GTGGTATTCT
Pmi04:1191  AGCCAGTTAT GGGGCCAAGC AAACGGAGCT TATGGCTACA ATTGAAGGAT TGCGTTTTGC TCTTGACATG GACGTATTTG ACTTGTGCCA TTCTAGAGAT GGACGCAAAA GAATGCATAA CTAGTATTCT
Pam_chr4:1  CGCCAGTTAT GGGGCCAAGC AAACAGAGCT TATGGCTACA ATTGAAGGAT TGCGTTTTGC TCTTGACATG GACGTATTTG ACTTGTGCCA TTCTAGAGAT GGACGCAAAA GAATGCATAA CTGATATTCT
            261                                                                                                                                           390
Pd04:11477  TACGACGGAG TAATGCAATG GAACATATAG TCTTTTACTT GAAGAAGCAA AATGCATATT AATTGAA--- -GAGTAGCTT TATGTCAATA GTCCCTAAGG AGCTGCAATA AGGTGGCACA GATCTTGGCT
Pp04:11125  TATGACGGAG TAATGCAATG GAACATATAG TCTTTTACTT GAAGAAGCAA AATGCATATT AAGTAAATTT AGAGTAGCTT TATGTCAATG GTCCCTAAGG ATCTGCAATA AGGTGGCACA CATCTTGGCT
Pka04:1145  TATGACGGAG TAATGCAATG GAACATATAG TCTTTTACTT GAAGAAGCAA AATGCATATT AAGTAAATTT AGAGTAGCTT TATATCAATG GTCCCTAAGG ATCTGCAATA AGGTGGCACA CATCTTGGCT
Pda04:1175  TACGACGGAC TAATGCAATG GAACATATAG TCTTTTACTT GAAGAAGCAA AATGCATATT AAGTGAATTT AGAGTAGCTT TATGTCAATG GTCCCTAAGA AGCTGCAATA AGGTGGCACA CATCTTGGCT
Pmi04:1191  TACGACGGAG TAATGCAATG GAACATATAG TCTTTTACTT GAAGAAGCAA AATGCATATT AAGTAAATTT AGAGTAGCTT TATGTCAATG GTCCCTAAGG ATCTGCAATA AGGTGGCACA CATCTTGGCT
Pam_chr4:1  TTTGGCGGAG TAATGCAATG GAACATATAG TCTTTTACTT CAAGAAGCAA AATGCATATT AAGTAAATTT AGAGTAGCTT TATGTCAATT GTCCCTAAGG AGCTGCAATA AGGTGGCACA CACCTTGGCT
            391                                                                                                                                           520
Pd04:11477  CAATTTGCAA TCAGCTTTAA TGATTTTGTT TCTTGGATTG AGGAAGCACA CCTACATGGT TGCTCTCATT TCTTTCTGCA GATGTACTCT ATTCTCGAGT TTTTGAATGA AATCTTGATT CTAAAATTAT
Pp04:11125  CAATTTGCAA TCAGCTTTAA TGATTTTGTT TCTTGGATTG AGGAAGCAC- -CTACATGGT TGCTCTCATT TCTT------ -----ACTCT CTTCTCGAGT TTTTGAATGA AATCTTGATT CTAAAATTAT
Pka04:1145  CAATTTGCAA TCAGCTTTAA TGATTTTGTT TCTTGGATTG AGGAAGCAC- -CTACATGGT TGCTCTCATG TCTTTATGCA GATGTACTCT CTTCTCGAGT TTTTGAATGA AATCTTGATT CTAAAATTAT
Pda04:1175  CAATTTGCAA TCAGCTTTAA TGATTTTGTT TCTTGGATTG AGGAAGCAC- -CTACATGGT TGCTCTCATT TCTTTCTGCA GACGTACTCT CTTCTCGAGT TTTTGAATGA AATCTTGATT CTAAAATTAT
Pmi04:1191  CAATTTGCAA TCAGCTTTAA TGATTTTGTT TCTTGGATTG AGGAAGCAC- -CTACATGGT TGCTCTCATT TCTTTCTGCA GATGTACTCT ATTCTCGAGT TTTTGAATGA AATCTTGATT CTAAAATTAT
Pam_chr4:1  CAATTTGCAA TTAGCTTTAA TGATTTTGTT TCTTGGATTG AGGAAGCAC- -CTACATGGT TGCACTC--- ---------- ---------- ------GAGT TTTTGAATGA AATCTTGATT CTAAAATTAT
            521                                                                                                                                           650
Pd04:11477  AAATAAAGAA TATAT----- ---------- -GATTTATTT ATGGTTTTCG TTAGATGAAC AATTAATTAA TGAAGATTAA T--------- ---------- --------AG ATATACTTTT TAATACCGAG
Pp04:11125  AAATAAAGAA TATATATATT ---------- --ATTTATTT ATGGTTTTCG TTAGATGAAC AATTAATTAA TGAAGATTAA TCCTCTATTT CGTTAGATGA AATGATTTAG ATATACTTTT TAATACCGAG
Pka04:1145  AAATAAAGAA TATATATATT ---------- --ATTTATTT ATGGTTTTCG TTAGATGAAC AATTAATTAA TGAAGATTAA TCCTCTATTT CGTTAGATGA AATGATTTAG ATATACTTTT TAATACCGAG
Pda04:1175  AAATAAAGAA TATATATATA T--------- -GATTTATTT ATGGTTTTCG TTAGATGAAC AATTAATTAA TGAAGATTAA T--------- ---------- --------AG ATATACTTTT TAATACCGAG
Pmi04:1191  AAATAAAGAA TATATATATA TATATATATA TGATTTATTT ATGGTTTTCG TTAGATGAAC AATTAATTAA TGAAGATTAA T--------- ---------- --------AG ATATACTTTT TAATACAGAG
Pam_chr4:1  AAATAATATA TTTAT----- ---------- ----TTATTT ATGGTTTTCG TAAGATGAAC AATTAATTAA TGAAGATTAA TCCTCTATTT CGTTAGATGA AATGATGTAG ATGTACTTTT TAATACCGAG
            651                                                                                                                                           780
Pd04:11477  GAAGCAGATA GGTCTCCAAT CGAAGTCAGT CATGCTAAAC CTCAAATTTT TTT-AAAAAG TATCACATTC ATAGAAAGAG GGAAGGAAAG CTGACAAAAT CTTTACACAA AAGAAGCTAA CTTTAATTTA
Pp04:11125  GAAGCAGATA GGTCTTCAAT CGAAGTCAGT CATGCTAAAC CTCAATTGTT TTT-CAAAAG TATCACATTC ATAGAAAGAG GGAGGGAAAG CTGACAAAAT CTTTATACAA AAGAAGCTAA CTTTAATTTA
Pka04:1145  GAAGCAGATA GGTCTTCAGT CGAAGTCAGT CATGCTAAAC CTCAATTTTT TTTTAAAAAG TATCACATTC ATAGAAAGAG GGAGGGAAAG CTGACAAAAT CTTTATACAA AAGAAGCTAA CTTTAATTTA
Pda04:1175  GAAGCAGATA GGTCTCCAAT CGAAGTCAGT CATGCTAAAC CTCAATTTTT TTT-TAAAAG TATCACATTC ATAGAAAGAG GGAGGGAAAG CTGACAAAAT CTTTATACAA AAGAAGCTAA CTTTAATTTA
Pmi04:1191  GAAGCAGATA GGTCTCCAAT CGAAGTCAGT CATGCTAAAC CTCAAATTTT TTT-AAAAAG TATCACATTC ATAGAAAGAG GGAGGGAAAG CTGACAAAAT CTTTATACAA AAGAAGCTAA CTTTAATTTA
Pam_chr4:1  GAAGCAGATA GGTCTCCAAT CGAAGTCAGT CATGCTAAAC CTCAAGAAAT TTA-AAAAAG TATCACATTC ATAGAAAGAA GGAGGGAAAG CTGACAAAAT CTTTATACAA AAGAAGCTAA CTTTAATTTA
            781                                                                                                                                           910
Pd04:11477  TATGTGGCCA GCTTGAAAGG TGCATCCGTG GCGTTGATAC TTCTCATCTA TTTGATACCA TGTATCTTTG CTCTAAAATG TGGCCAATTT TTTGTCCTCA TTTCAAAATT TGGCAAGTGA TTGTACTTGA
Pp04:11125  TATGTGGCCA GCTTGAAAGG TGCATCCGTG GCGTTGATAC TTCTCATCTA TTTGATACCA TGTATCTTTG CTCTAAAAAG TGGCCAATTT TTTGTCCTCA TTTCAAAATT TGGCAAGTGA TTGTACTTGA
Pka04:1145  TATCTGGCCA GCTTGAAAGG TGCATCCGTG GCGTTGATAC TTCTCATCTA TTTGATACCA TGTATCTTTG CTCTAAAAAG TGGCCAATTT TTTGTCCTCA TTTCAAAATT TGGCAAGTGA TCGTACTTGA
Pda04:1175  TATGTGGCCA GCTTGAAAGG TGCATCCGTG GCGTTGATAC TTCTCATCTA TTTGATACCA TGTATCTTTG CTCTAAAAAG TGGCCAATTT TTTGTCCTCA TTTCAAAATT TGGCAAGTGA TTGTACTTGA
Pmi04:1191  TATGTGGCCA GCTTGAAAGG TGCATCCGTG GCGTTGATAC TTCTCATCTA TTTGATACCA TGTATCTTTG CTCTAAAAAG TGGCCAATTT TTTGTCCTCG TTTCAAAATT TGGCAAGTGA TTGTACTTGA
Pam_chr4:1  TTTATGGCCA GCTTGAAAGG TGCATCCATG GCGTTGATTC TTCTCATCTA TTTGATACCA TGTATCTTTG CTCTAAAAAG TGGCCAATTA TTTGTCCTCA TT-CAAAATT TGGCAAGTGA TTGTACTTGA
            911                                                                                                                                           1040
Pd04:11477  CAAGAAATTA ATATTTCTAA TTTGGCTTTA AAACGACAAT TAGTGAAAGC AACCCCCACC TCACAATCTG TCTCGTTCTC TGTATAAGGC TAAGCAAGAT TTGGCTACTA ACACCACCTT GATTGGCTTT
Pp04:11125  CAAGAAATTA ATATTTCCAA TTTGGCTTTA AAACGACAAT TAGTGAAAGC AACCCCCAAC TCACAATCTG TCTCGTTCTC TGTATAAGGC TAAGCAAGAT TTGGCTACTA ACACCACCTT GATTGGCTTT
Pka04:1145  CAAGAAATTA ATATTTCCAA TTTGGCTTTA AAACGACAAT TAGTGAAAGC AACCCCCAAC TCACAATCTG TCTCGTTCTC TGTATAAGGC TAAGCAAGAT TTGGCTACTA ACACCACCTT GATTGGCTTT
Pda04:1175  CAAGAAATTA ATATTTCCAA TTTGGCTTTA AAACGACAAT TAGTGAAAGC AACCCCCAAC TCACAATCTG TCTCGTTCTC TGTATAAGGC TAAGCAAGAT TTGGCTACTA ACACCACCTT GATTGGCTTT
Pmi04:1191  CAAGAAATTA ATATTTCCAA TTTGGCTTTA AAACGACAAT TAGTGAAAGC AACCCCCAAC TCACAATCTG TCTCGTTCTC TGTATAAGGC TAAGCAAGAT TTGGCTACTA ACACCACCTT GATTGGCTTT
Pam_chr4:1  CAAGAAATTA ATATTTCCAA TTTGGCTTTA AAACGACAAT TAGTGAAAGC AACCCCCAAC TCACAATCTG TCTCGTTCTC TGTATAAGGC TAAGCAAGAT TTGGCCACCA ACACCTCCTT GATTGGCTTT
            1041                                                                                                                                          1170
Pd04:11477  GTTAAGCTCT CTTCCTTGAC TTATCAACAC ACGAAAGATA TCTTTAAGCA CTAATGTAAT CCTTCTCCAA TGAGGGATAT ACTCAACAAT TTTTTTGTAT GTTACTATTT AGGGGAGAAC ATATTTT-CA
Pp04:11125  GTTAAGCTCT CTTCCTTGAC TTATCAACAC ACGAAAGATA TCTTTAAGCA CTAATGCAAT CCTTCTCCAA TGAGGGATAT ACTCAACAAT TTTTTTGTAT GTTACTATTT AGGGGAGAAC ATATTTTTCA
Pka04:1145  GTTAAGCTCT CTTCCTTGAC TTATCAACAC ACGAAAGATA TCTTTAAGCA CTAATGTAAT CCTTCTCCAA TGAGGGATAT ACTCAACAAT TTTTTTGTAT GTTACTATTT AGGGGTGATC ATATTTTTCA
Pda04:1175  GTTAAGCTCT CTTCCTTGAC TTATCAACAC ACGAAAGATA TCTTTAAGCA CTAATGTAAT CCTTCTCCAA TGAGGGATAT ACTCAACAAT TTTTTTGTAT GTTACTATTT AGGGGAGATC ATATTTTTCA
Pmi04:1191  GTTAAGCTCT CTTCCTTGAC TTATCAACAC ACGAAAGATA TCTTTAAGCA CTAATGTAAT CCTTCTCCAA TGAGGGATAT ACTCAACAAT TTTTTTGTAT GTTACTATTT AGGGGAGAAC ATATTTTTCA
Pam_chr4:1  GTTAAGCTCT CTTCCTTGAC TTATCGACAC ACGAAAGATA TCTTTAAGCA CTAATGTAAT CCTTCTCCAA TGAGGGATAT ACTCAACAAT TTTTT-GTAT GTTACAATTT ATGGGAGAAC ATATTTTTCA
            1171                                                                                                                                          1300
Pd04:11477  TTGTAGTTTT TGTACTTATA TTGACTTAAT GGTGGTGACC ACTTGATTAA TACTAATCCG TAAACAAAAC CAGATGGAGG GCCAGCATTT TTGAATTTTT GAGTCAAGAA ACAAACATTA CAAAGAAGAT
Pp04:11125  TTGTAGTTTT TGTACTTATA TTGACTTAAT GGTGGTGACC ACTTGATTAA TACTAATCCG TAAACAAAAC CAGATGGAGG GCCAGCATTT TTGAATTTTT GAGTCAAGAA ACAAACATTA CAAAGAAGAT
Pka04:1145  TTGTAGTTTT TGTACTTATA TTGACTTAAT GGTGGTGACC ACTTGATTAA TACTAATCCG TAAACAAAAC CAGATGGAGG GCCAGCATTT TTGAATTTTT GAGTCAAGAA ACAAACATTA CAAAGAAGAT
Pda04:1175  TTGTAGTTTT TGTACTTATA TTGACTTAAT GGTGGTGACC ACTTGATTAA TACTAATCCG TAAACAAAAC CAGATGGAGG GCCAGCATTT TTGAATTTTT GAGTCAAGAA ACAAACATTA CAAAGAAGAT
Pmi04:1191  TTGTAGTTTT TGTACTTATA TTGACTTAAT GGTGGTGACC ACTTGATTAA TACTAATCCG TAAACAAAAC CAGATGGAGG GCCAGCATTT TTTAATTTTT GAGTCAAGAA ACAAACATTA CAAAGAAGAT
Pam_chr4:1  TTGT---TTG TGTACTTATG TTGACTTAAT GGTGGTGACC ACTTGATTAA TGCTAATCCG TAAACAAAAC CAGATGGAGG GCCAGCATTT TTGAATTTTT GAGTCAAGAA ACAAACATTA CAAAGAAGAT
            1301                                                                                                                                          1430
Pd04:11477  CCTCGAGGGA TATTTACACC ATATAAATCA CAATATATA- -CTCTATGTA AAACATTCGT TGGAAAATTT TGAAACCAAA TACACGTAGC TGAAGAAGCA TGATCACGTT TAAACATGCT TTTAAATATA
Pp04:11125  CCTCGAGGGA TATTTACACC ATATAAATCA CAATATATAT ACTCTATGTA AAACATTCAT TGGAAAATTT CGAAACCAAA TACACGTAGC TGAAGAAACA TGATCACGTT TAAACATGCT TTTAAATATA
Pka04:1145  CCTCGAGGGA TATTTACACC ATATAAATCA CAATATATAT ACTCTATGTA AAACATTCAT TGGAAAATTT CGAAACCAAA TATACGTAGC TGAAGAAGCA TGATCACGTT TAAACACGCT TTTAAATATA
Pda04:1175  CCTCGAGGGA TATTTACACC ATATAAATCA CAATATATAT ACTCTATGTA AAACATTCAT TGGAAAATTT CGAAACCAAA TACACGTAGC TGAAGAAGCA TGATCACGTT TAAACATGCT TTTAAATATA
Pmi04:1191  CCTCGAGGGA TATTTACACC ATATAAATCA CAATATATAT ACTCTATGTA AAACATTCGT TGGAAAATTT CGAAACCAAA TACACGTAGC TGAAGAAGCA TGATCACGTT TAAACATGCT TTTAAATATA
Pam_chr4:1  CCTCGAGGGA TATGTACACC ATATAAATCA CAATCTACTC TATATATGTA AAACATTCAT TGGAAAATTT CGAAACCAAA TACACGTAGC TGAAGAAGCA ---------- --------CT TTTAAATGTA
            1431                                                                                                                                          1560
Pd04:11477  CATCAAATTT AAACATCAAG GGTCAAATAA AATTTCACTC AAA-CAACAT GAATGTAAAA TGTAATTTAC AAATATTTAT ATTTAATATA TGTATGCATA CTGCAAATAT AGTATAATGT CTCTTGTTTA
Pp04:11125  CATAAAATTT AAACATCAAG GCTCAAATAA AATTATACTC AAA-CAACAT GAATGTAAAA TGTAATTTAC AAATATTTAT ATTTA-TATA TGTATGCATA CTGCAAATAT AGTATAATGT CTCTTGTTTA
Pka04:1145  CATCAAATTT AAACATCAAG GGTCAAATAA AATTTCACTC AAA-CAACAT GAATGTAAAA TGTAATTTAC AAATATTTAT ATTTA-TATA TGTATGCATA CTGCAAATAT AGTATAATGT CTCTTGTTTA
Pda04:1175  CATCAAATTT AAACATCAAG GGTCAAATAA AATTTCACTC AAA-CAACAT GAATGTAAAA TGTAATTCAC AAATATTTAT ATTTA-TGTG TGTATGCATA CTGCATATAT AGTATAATGT CTCTTGTTTA
Pmi04:1191  CATCAAATTT AAACATCAAG GGTCAAATAA AATTTCACTC AAAACAACAT GAATGTAAAA TGTAATTTAC AAATATTTAT ATTTA-TATA TGTATGCATA CTGCAAATAT AGTATAATGT CTCTTGTTTA
Pam_chr4:1  CATCAAATTT AAACATCAGG GGTCAAATAA AATTTCACTC AAA-CAACAT GAATGTAAAA TGTAATTCAC AAATATTTAT ATTTA-TATA TGTATGCATA CTGCATATAT AGTATAATGT CTCTTGTTTA
            1561                                                                                                                                          1690
Pd04:11477  TAGATGTATG AAAGTGTGTG ACATATCAAC ATAAATACAA TTTATACATG GAAATTAGCT GTCGTGACAA TGCGATTTCG ATCGAGCTGT CTCTCTCCAA ATACTGAAAG CCAAATCGCA GAAATGAATT
Pp04:11125  TAGATGTATG AAAGTGTGTG ACATATCAAC ATAAATACAA TTTATACATG GAAATTAGCT GTCGTGACAA TGGGATTTCG ATCGAGCTGT CTCTCTCCAA ATACTGAAAG CCAAATCACA GAAATGAATT
Pka04:1145  TAGATGTATG AAAGTGTGTG ACATATCAAC ATAAATACAA TTTATACATG GAAATTAGCT GTCGTGACAA TGGGATTTCG ATCGAGCTGT CTCTCTCCAA ATACTGAAAG CCAAATCACA GAAATGAATT
Pda04:1175  TAGATGTATG AAAGTGTGTG ACATATCAAC ATAAATACAA TTTATACATG GAAATTAGCT GTCGTGACAA TGGGATTTCG ATCGAGCTGT CTCTCTCCAA ATACTGAAAG CCAAATCACA GAAATGAATT
Pmi04:1191  TAGATGTATG AAAGTGTGTG ACATATCAAC ATAAATACAA TTTATACATG GAAATTAGCT GTCGTGACAA TGGGATTTCG ATCGAGCTGT CTCTCTCCAA ATACTGAAAG CCAAATCACA GAAATGAATT
Pam_chr4:1  TACATGTATG AAAGTGTG-- ACATATCAAC ATAAATACAA TTTATACATG GAAATTAGCT GTCATGACAA TGGGATTTCG ATCGAGCTGT CTCTCTCCAA ATACTGAAAG CCAAATCACA GAAATGAATT
            1691                                                                                                                                          1820
Pd04:11477  ATTGTCCAAA TTAATCAGAC CCAACTGTCA TTATAACAGG CTGTGATTTC TTAAGATTAA CGTGATATGA CATGCTAAGA GATCGTAGGA TATTTATCAG GAAGATGACA CATTTTGTT- GATTGGATAA
Pp04:11125  ATTGTCCAAA TTAATCAGAC CCAACTGTCA TTATAACAGG CTGTGATTTC TTAAGATTAA CGTGATATGA CATGCTAAGA GATCGTAGGA TATTTATCAG GAAGATGACA CATTTTGTT- GATTGGATAA
Pka04:1145  ATTGTCCAAA TTAATCAGAC CCAACTGTCA TTATAACAGG CTGTGATTTC TTAAGATTAA CATGATATGA CATGCTAAGA GATCGTAGGA TATTTATCAG GAAGATGACA CATTTTGTTT GATTGGATAA
Pda04:1175  ATTGTCCAAA TTAATCAGAC CCAACTGTCA TTATAACAGG CTGTGATTTC TTAAGATTAA CGTGATATGA CATGCTAAGA GATCGTAGGA TATTTATCAG GAAGATGACA CATTTTGTT- GATTGGATAA
Pmi04:1191  ATTGTCCAAA TTAATCAGAC CCAACTGTCA TTATAACAGG CTGTGATTTC TTAAGATTAA CGTGATATGA CATGCTAAGA GATCGTAGGA TATTTATCAG GAAGATGACA CATTTTGTT- GATTGGATAA
Pam_chr4:1  ATTGTCCAAA TTAATCAGAC CCAACTGTCA TTATAATAGG CTGTGATTTC TTAAGATTAA CGTGATATGA CATGCTAAGA GATCGTAGGA TTTTTATCAG GAAGATGACA CATT--GTT- GATTGGATAA
            1821                                                                                                                                          1950
Pd04:11477  AAGATTTTAT ATGGAGAAAA AGAGAAAATA CACAAGAGTT TGTGAGACTA ATCCTTCTAT TTTAAGCTCA CAATTCTGGA ATTGATTTGA TTGATCGAAA TAATATCGTC TCCTTTCAAC AAAGAAATTT
Pp04:11125  AAGATTTTAT ATGGAGAAAA AGAGAAAATA CACAAGAGTT TGTGAGACTA ATCCTTCTAT TTTAAGCTCA CAATTCTGGA ATTGATTTGA TTGATCGAAA TAATATCGTC TCCTTTCAAC AAAGAAATTT
Pka04:1145  AAGATTTTAT ATGGAGAAAA AGAGAAAATA CACAAGAGTT TGTGAGACTA ATCCTTCTAT TTTAAGCTCA CAATTCTGGA ATTGATTTGA TTGATCGAAA TAATATCGTC TCCTTTCAAC AAAGAAATTT
Pda04:1175  AAGATTTTAT ATGGAGAAAA AGAGAAAATA CACAAGAGTT TGTGAGACTA ATCCTTCTAT TTTAAGCTCA CAATTCTTGA AGTGATTTGA TTGATCGAAA TAATATCGTC TCCTTTCAAC AAAGAAATTT
Pmi04:1191  AAGATTTTAT ATGGAGAAAA AGAGAAAATA CACAAGAGTT TGTGAGACTA ATCCTTCTAT TTTAAGCTCA CAATTCTGGA ATTGATTTGA TTGATCGAAA TAATATCGTC TCCTTTCAAC AAAGAAATTT
Pam_chr4:1  AAGATTTTAT ATGGAGAAAA AGAGAAAATA CACAAGAGTT TGTGAGACTA ATCCTTCTAT TTTAAGCTCA CAATTCTGGA AGTGATTTGA TTGATCGAAA TAATATCGTT TCCTTTCAAC AAAGAATTTT
            1951                                                                                                                   2053
Pd04:11477  TAAAAA--AA TAATAATTAG GACACTTATA ATTTGTATCG TTGCATGGTG AGCACTTACG GATAATGTTG AGAGAATTGG GGTGTTTACT AAGAGTGGTG TT
Pp04:11125  TTTAAA-AAA TAAAAATTAG GGCACTTATA ATTTGTATCG TTGCATGGTG AGTACTTATG GATAATGTTG AGAGAATTGG GGTGTTTATT AAGAGTGGTG TT
Pka04:1145  AA-AAA-TAA TAATAATTAG GGCACTTATA ATTTGTATCA TTGCATGGTG AGTACTTATG GATAATGTTG AGAGAATTGG GGTGTTTATT AAGAGTGGTG TTA
Pda04:1175  TAAAAA-AAA TAAAAATTAG GGCACTTATA ATTTGTATCG TTGCATGGTG AGTACTTATG GATAATGTAG AGAGAATTAG GGTGTTTATT AAGAGTGGTG T
Pmi04:1191  AAAAAATAAA TAAATATTAG GGCACTTATA ATTTGTATCG TTGCATGGTG AGTACTTATG GATAATGTTG AGAGAATTGG GGTGTTTATT AAGAATGGTG TT
Pam_chr4:1  TTTTTTAAAA TAAAAATTAG GGCACTTATA ATTTGTATCG TTGCATGGTG AATACTTATG GATAATTTTG AGAGAATTGG GGTGTTTATT AAGAGT
```

Supplementary Figure S7. Association between SNP Peach_AO_0443007 and MD. Boxplots show MD values for genotypes AA, AG and GG. Differences among genotypes were tested by ANOVA and Tukey's HSD.

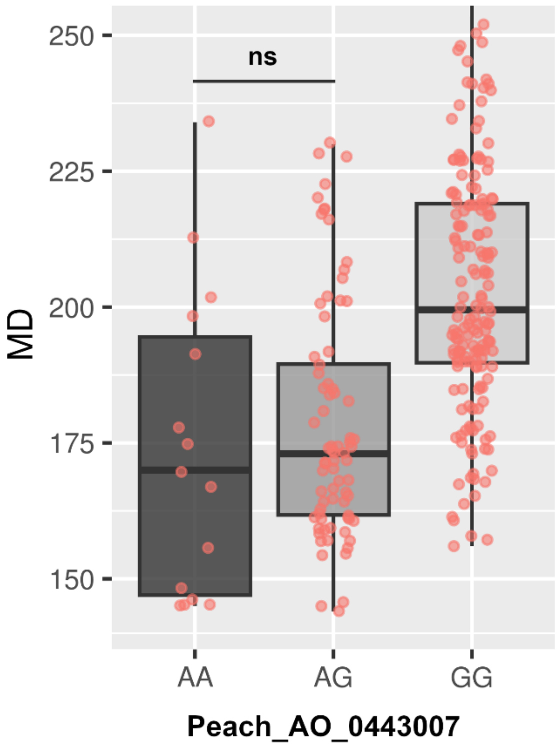

Supplement: Supplementary file 1 [file erag210_supplementary_data.zip › JEXBOT317664-file001.pdf]
